# Misinformedness about the European Union and the Preference to Vote to Leave or Remain

**DOI:** 10.1111/jcms.13316

**Published:** 2022-01-24

**Authors:** Julia Partheymüller, Sylvia Kritzinger, Carolina Plescia

**Affiliations:** ^1^ Vienna Centre for Electoral Research University of Vienna Vienna; ^2^ Department of Government University of Vienna Vienna

**Keywords:** misinformedness, European Union, referendum, political knowledge, confidence in knowledge

## Abstract

European politicians have become increasingly concerned about the possible distorting effects of citizens not only being *un*informed, but systematically *mis*informed about the European Union (EU). Against this background, this study assesses the role of EU knowledge in shaping the preference to vote to leave or remain in a (hypothetical) referendum on EU membership using cross‐national survey data that were collected simultaneously in eight EU countries during the run‐up to the 2019 EP elections. The surveys included a newly designed item battery of EU knowledge capturing both the accuracy as well as confidence in knowledge of the respondents. The results show that misinformedness is associated with a preference to leave the EU, the uninformed citizens tend to be undecided or not intending to vote, while the well‐informed prefer to remain. Overall, our findings contribute to the ongoing debates about the role of misinformation in politics.

## Introduction

In recent years, European politicians and political observers have become increasingly wary about voters being misinformed about politics. Most notably, the Brexit referendum played a critical role in triggering such worries. In the referendum on 23 June 2016 a majority of the British people voted to leave the European Union (EU), a supranational institution it had joined 43 years earlier in 1973. The outcome of the Brexit referendum sent financial and political shockwaves around the world generating heated debate about what has led UK citizens to vote to leave (Hobolt, 2016; Clarke *et al*., 2017). In this regard, many discussions arose on citizens' informedness about the EU. Scholarly research as well as pundit discussions became also focused on the risk of a potential ‘domino effect’ – that is, that similar referendums in other member states may lead to the same outcome, thus posing a severe threat to the existence of the EU itself (for example, Hobolt, 2016).

Indeed, a necessary condition for the proper functioning of democracies is that citizens are well informed about the decisions at stake (Banducci *et al*., 2017). Yet, mere uninformedness might not even be particularly harmful. Political ignorance – that is, the absence of information – is mostly thought to result in rather arbitrary individual errors that cancel out in the electorate at large (Converse, 1990; Page and Shapiro, 1992; Stimson, 2015). In contrast, misinformed people may take political action on the basis of systematically incorrect information, becoming what Hochschild and Einstein (2015) call the ‘active misinformed’. As a result, voters' misinformedness has a potential of leading to a systematic distortion of outcomes.

Given the complex institutional structure of the EU and the tendency of domestic politicians to engage in a ‘blame game’ with Brussels, EU‐related referendums are particularly likely to be affected by misinformedness as the issues are often cross‐cutting to usual party lines (LeDuc, 2009). Unlike in elections where voters choose their representatives, common heuristics (Popkin, 1991) such as party identification cannot be applied in the same way and protect against the distorting effects of misperceptions in direct‐democratic decisions.

In this paper, we therefore bring together two strands of literature – both the literature on misinformedness and EU membership preferences. Relying on the two‐dimensional conception of political knowledge introduced by Kuklinski *et al*. (1998, 2000) who have emphasised the importance of confidence in one's own knowledge as a factor shaping public opinion, we designed a new item battery to measure EU informedness capturing not only the factual accuracy of beliefs regarding EU institutions and policies, but also the respondents' confidence in their knowledge about the EU. Based on this novel measure, we can distinguish not only between well‐informed and uninformed citizens, but also focus on misinformed voters. Studying the underlying cognitions of hypothetical preferences has the advantage that we can observe the ‘naturally occurring’ associations between beliefs and preferences to leave or remain in the EU in the absence of an actual ongoing campaign where active mobilization and counter‐mobilization will exert an additional influence on preference formation (Hobolt and De Vries, 2016). Using original survey data from eight EU member states (Austria, Denmark, France, Germany, Hungary, Italy, Poland, Spain) collected in the context of the 2019 European Parliament elections, we demonstrate that misinformed citizens are characterized by distinct preferences regarding their country's EU membership, which sets them apart from the well‐informed and uninformed. Our findings underline that the concept of confidence in knowledge is relevant for our understanding of public opinion.

## I The Two‐Dimensional Structure of Knowledge and Types of Informedness

**Figure 1:**
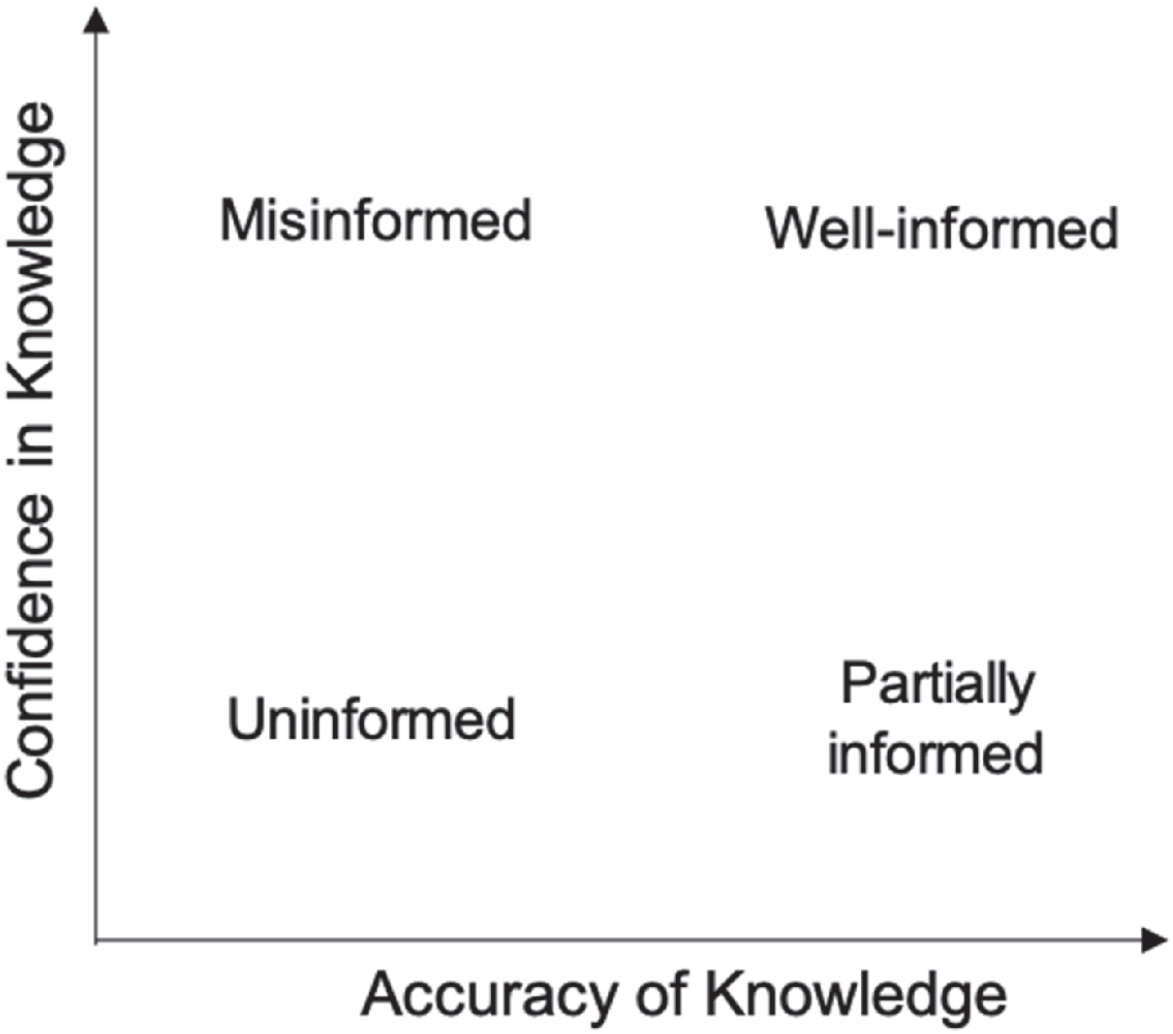
The Two Dimensions of Political Knowledge and Four Types of Informedness *Note*: Own diagram based on Lee and Matsuo (2018).

We rely on the two‐dimensional conception of knowledge as introduced by Kuklinski *et al*. (1998, 2000) and as shown in Figure 1. The first dimension is the accuracy of knowledge. Accuracy has often been simply equated with knowledge as such, leaving the second dimension which is confidence in knowledge aside. The accuracy of political knowledge can be defined as a continuum ranging from low to high amounts of factually correct information about politics that is stored in long‐term memory (Delli Carpini and Keeter, 1996). At the individual level, the accuracy of political knowledge has been explained by well‐known antecedents such as political interest, education, and exposure to political news in the media (for example, Luskin, 1990; Delli Carpini and Keeter, 1996; Kwak, 1999).

The second dimension, far less studied, is confidence in knowledge. Confidence represents ‘an individual's perceptions of his or her knowledge’ (Radecki and Jaccard, 1995, p.107). As such, confidence in knowledge can be thought of as a meta‐cognition of how certain a person feels about the accuracy of his or her beliefs reflecting the availability and accessibility of information stored in memory upon retrieval. In line with this notion, Lee and Matsuo (2018) demonstrate that greater confidence is associated with lower response time, suggesting greater cognitive availability and accessibility of responses about which respondents felt confident that they are correct.

The existing literature has also shown that the two dimensions – accuracy and confidence – cannot only be distinguished analytically, but also empirically each with unique antecedents and its own measurement (Park *et al*., 1994; Radecki and Jaccard, 1995). While a common finding in psychological research is that confidence is positively related to accuracy (namely that citizens believe they know more when they actually do know more) (for example, Allwood and Montgomery, 1987; Griffin and Tversky, 1992), Lee and Matsuo (2018) illustrate that there is only a mild positive correlation between measures of accuracy and confidence, and that education is positively correlated with accuracy in political knowledge but not related to confidence in knowledge.

Kuklinski *et al*. (2000, pp. 794–5) mention three mental processes documented by social cognitive psychology, which may lead to overconfidence: (1) citizens are likely to draw inferences based on their observations, (2) they try to make inferences that are consistent with the information already stored in their minds, and (3) those inferences become stored as ‘hard data’ that are considered to be equally valid than facts learned based on actual evidence. As a result of these cognitive mechanisms, some people tend to be overconfident. Meanwhile, overconfidence is very often associated with gender with men overestimating their abilities (Kling *et al*., 1999; Soll and Klayman, 2004; Niederle and Vesterlund, 2011), but also with age with older persons being more likely to show patterns of overconfidence in their political reasoning (Ortoleva and Snowberg, 2015). Also, people with higher education seem to be more confident (Baumeister *et al*., 2003), as do people holding more extreme political positions (Ortoleva and Snowberg, 2015).

Based on the two dimensions of knowledge, citizens can be broadly grouped into different types of informedness. We follow Lee and Matsuo (2018) and speak of citizens as well‐informed, uninformed, partially informed or misinformed based on the following constellations of accuracy and confidence in knowledge (see Figure 1): (1) well‐informed citizens possess accurate information and are confident, (2) the misinformed hold mostly inaccurate information but are nevertheless fairly confident that they got the facts right, (3) the uninformed hold inaccurate information and lack confidence, and (4) the partially informed are rather high in accuracy but low in confidence. For us, in particular, the interplay between accuracy and confidence will be relevant in the following.

## II The Relationship between EU Knowledge and EU Membership Preferences

Based on normative notions of democracy, it is desirable that citizens possess an enlightened understanding of politics (Dahl, 1989) and use this to decide what serves their interests best. A certain level of accurate political information is thought to be fundamentally important for citizens' ability to match their preferences and interests with specific public issues and to promote political participation (for example, Delli Carpini and Keeter, 1996). Thus, the notion prevails that a lack of factual information tends to misguide citizens in their policy preferences and behaviours (Kuklinski *et al*., 2000) and biases the shape of collective opinion (Althaus, 2003).

But accuracy is not all that matters. Confidence in knowledge influences politically relevant behaviours as well. For example, confidence in knowledge tends to make people less likely to search for information (Dancey and Sheagley, 2013), and much more likely to rely on cognitive shortcuts such as their prior beliefs and preferences (Koch, 2003). What is more, voters possessing high confidence are more likely to believe that they have all the knowledge they need to make a decision (Weary and Jacobson, 1997; Tiedens and Linton, 2001). Low accuracy in pair with high confidence may thus mislead citizens' political choices and distort their opinions at much higher rates – leading to false evaluations of events, processes or threats – rather than when they just hold inaccurate information alone (Lewandowsky *et al*., 2005; Flynn *et al*., 2017).

Various circumstances contribute to European citizens being misinformed about the EU. Courts and legal scholars have described the EU with regard to its institutional design as a creation *sui generis*, with little historical or international precedent for citizens to compare it to. Democratic processes at the EU level are fairly complex with some scholars pointing to its democratic deficit (Follesdal and Hix, 2006). Despite reforms and the strengthened role of the European Parliament, policy‐making at the EU level remains highly complex. The interplay and power‐sharing among various institutions at the EU level and the nation states seems to provide ample opportunity for misunderstandings of how democracy in the EU actually functions and what the role of the member states is. It is thus perhaps not surprising that knowledge of the EU is consistently found to be lower compared to knowledge of national or local level of politics (for example, Rapeli, 2014).

Previous research has also shown that national politicians tend to engage in a ‘blame game’ with Brussels, obfuscating their own role in the decision‐making process at the EU level, portraying actions by the EU as illegitimate external interference, and characterizing the EU as an overly powerful bureaucratic and wasteful entity (for example, Schlipphak and Treib, 2017). In recent years, with the EU facing several crises (Baglioni and Hurrelmann, 2016) it often was portrayed in dramatised ways by the news media. In the face of the actual level of complexity, misleading domestic portrayal of EU politics, and repeatedly occurring dramatised crisis events, it does not seem surprising that some citizens may misperceive the working and competences of the EU resulting in distorted evaluations thereof.

### Hypotheses

What expectations follow based on these considerations for opinion formation of preferences in a (hypothetical) referendum on EU membership? We derive as a first expectation that the well‐informed will be more likely to prefer to remain in the EU than those with views being distorted by common portrayals of the EU as an illegitimate actor. This does not necessarily mean that ‘to know it, is to love it’ (Karp *et al*., 2003, p. 271), but suggests that the well‐informed will show somewhat greater support than those drawing inferences based on common distorted portrayals. Previous research provides support for this expectation. Empirically, for instance, Inglehart (1970, p. 54) finds that ‘cognitive mobilization [is] the most powerful predictor of pro‐European attitudes’ confirming that ‘all information about [EU] integration promotes [EU] support’ (Gabel, 1998, p. 335). Likewise, Janssen (1991, p. 459) shows that ‘a higher level of political skills is indeed strongly related to a more supportive attitude towards integration and EC membership’ and Anderson (1998, p. 589) finds that ‘interest in EU politics was the most significant and consistently important variable affecting support for EU membership’. Following the theory of cognitive mobilization (Inglehart, 1970), the supposed mechanism behind this association is that well‐informed citizens will overall feel more familiar with EU matters and will hence perceive the EU as less threatening. Based on that, we can infer that those holding accurate beliefs with confidence are the most likely to support the membership of their country in the EU.

This leads to our first hypothesis:

Hypothesis 1 High accuracy of knowledge in conjunction with high levels of confidence in knowledge about the EU is positively related to voting to remain in a hypothetical referendum on EU membership.

Secondly, we expect the misinformed to be the most likely to display a preference of voting to leave. Previous research has quite consistently documented an association between misinformedness, anti‐establishment attitudes and voting behaviour. For instance, van Prooijen and Krouwel (2019) found that low knowledge but high confidence about a specific political event tend to predict an anti‐establishment vote. This is confirmed by van Kessel *et al*. (2021) who show that misinformedness relates positively to support for right‐wing populist parties.

The psychological mechanisms behind this association are not fully known. However, some research suggests that perceptions of threat may play a role as also implied by the theory of cognitive mobilization. As argued above, citizens who lack accurate information about the EU may perceive the EU, which is based on blame‐portrayals and dramatised crisis events as threatening, creating the desire for seemingly simple solutions such as leaving the EU altogether. In a similar vein, some research suggests that the psychological link between an anti‐establishment vote and support for right‐wing populism may in part be due to the association between anti‐establishment thinking and attractiveness to conspiracy beliefs (Castanho Silva *et al*., 2017), which have also been linked to perceptions of threat and times of uncertainty (van Prooijen and Douglas, 2018).

Apart from that, the complexity of EU politics may matter. Facing overwhelming complexity, accurate information can be hard to come by and citizens are often faced with the challenge of verifying claims of unknown veracity. With the hard data being essentially inaccessible, people tend to ‘fill in the blanks’ (Kuklinski *et al*., 2000, p.794) drawing their own inferences based on their observations from everyday life generating simple, subjectively consistent narratives that reduce real‐world complexity. It is known that the stronger the initial opinion, the less likely one is to correct oneself and remains open to bias and inaccurate information (Cromby, 2019). This means that inaccurate information is likely to be fuelled by further inaccurate information increasing confidence in wrongly held beliefs against the EU fuelling even greater Euroscepticism (De Vreese, 2007). What further adds to the prevalence of misinformedness is that some people tend to feel overconfident and mistakenly treat inferences as hard data (Kuklinski *et al*., 2000), even when they arrived at those (false) conclusions based on inaccurate information or biased information‐processing. These theoretical considerations lead to our next hypothesis:

Hypothesis 2 Low accuracy of knowledge in conjunction with high levels of confidence in knowledge about the EU is positively related to voting to leave in a hypothetical referendum on EU membership.

Finally, it follows that citizens low on confidence in knowledge are less likely to make use of their beliefs when forming judgments and deciding on political actions. This has been demonstrated, for instance, by research on the impact of uncertainty in perceptions of issue positions: Citizens who feel uncertain about the positions of political parties are less likely to base their judgment and voting decisions on them (Alvarez and Franklin, 1994). Confidence is also strongly related with indifference and ambivalent views regarding politics and political candidates. Existing studies indicate that indifference and ambivalence reduce citizens' motivation to be politically active with uninformed voters overall less likely to vote or engage politically (for example, Huckfeldt *et al*., 2004; Mutz, 2006). This point is also supported by Lee and Matsuo (2018) who show that confidence relates to the frequency of political discussions, which is an important antecedent of forming an opinion and participation in politics. As a result, citizens with low confidence in knowledge – the uninformed and partially informed citizens – may decide not to turn out to vote, or remain rather undecided. From this follows our last hypothesis:

Hypothesis 3 Regardless of the accuracy of knowledge, low confidence in knowledge about the EU is positively related to being undecided about remain or leave, or choosing to abstain in a hypothetical referendum on EU membership.

## III Data and Methods

To evaluate the impact of accuracy of and confidence in EU knowledge on the evaluation of one's country's future in the EU, we make use of the data gathered by the RECONNECT and AUTNES project during the 2019 EP elections. As part of these projects, surveys were conducted simultaneously in eight EU countries, namely Austria, Denmark, France, Germany, Hungary, Italy, Poland and Spain (Aichholzer *et al*., 2020; Plescia *et al*., 2020). The country sample covers a variety of EU democracies, including old and new member states as well as countries from East and West and North and South of Europe. The country sample also spans considerably different contexts in terms of long‐standing attitudes towards the EU (Hobolt and De Vries, 2016; Kritzinger *et al*., 2020).

While the original study included two survey waves, for this paper, we will make exclusive use of the pre‐election data that was gathered in April 2019 since this wave included all relevant measures of both our dependent and independent variables. The data were collected as quota samples via online access panels, with quotas closely mirroring the population distributions of key demographics, namely age, gender, education and region. In all analyses, in addition, post‐stratification weights were applied to match population targets.

The dependent variable in our analysis is the question of how respondents would vote in a hypothetical referendum. We study vote choice in a hypothetical referendum on the EU which means that we will be able to focus on the impact of our main independent variable – that is, citizens' informedness on vote choice – net of the influence of the role of elites in shaping the information environment and campaign effects taking place during a real referendum campaign. 1 The question wording is as follows: ‘If there was a referendum about staying or leaving the EU in [COUNTRY], how would you decide?’. The answering options are ‘[COUNTRY] staying in the EU’, ‘[COUNTRY] leaving the EU’, ‘I am undecided’, and ‘I would not vote’. We collapse the undecided and non‐voters into one category as we have the same hypothesis for these two categories. As a result, our dependent variable groups respondents into three categories (Remain, Leave, and Undecided/Non‐Voter).

With regards to our independent variables, the surveys included a battery of knowledge questions on the EU similar to those usually asked in other Europe‐wide studies. The five questions covered, in particular, the major institutions of the EU involved in the policy process at the European level such as the European Parliament, the European Commission, and the Council of the European Union. The battery also included one question on EU policies and the budget. These questions constitute conventional measures of EU politics since they refer to the functioning of the EU political system and the actors involved, and are all relevant to EU politics as well as the daily lives of EU citizens. Appendix A includes the full question wording. All knowledge questions can be considered ideologically neutral since they are not necessarily more or less ideologically convenient for respondents preferring their country to remain an EU member or rather to leave the EU (our dependent variable). 2 Further below we discuss robustness checks showing that the results are robust to the use of partial subsets of the knowledge items.

Each knowledge question offered four answering options, with one being correct and three being false. Respondents were instructed to give their best guess if they did not know the answer. This means that there was no explicit ‘Do not Know’ response option available to respondents. This is first, to avoid that unequal propensities to opt for a ‘Do not Know’ answer affect the results (Mondak and Creel Davis, 2001), and second, to accommodate for the substantial gender differences in the use of such an answer category (Fraile, 2013). Instead, after answering each of the knowledge questions, respondents are asked in a follow‐up question to indicate how certain they are that their answer is correct on a 11‐point scale from 0 to 10, capturing the respondents' confidence in knowledge in the same way as suggested by previous research evaluating the role of misinformedness (Kuklinski *et al*., 2000; Lee and Matsuo, 2018).

**Figure 2:**
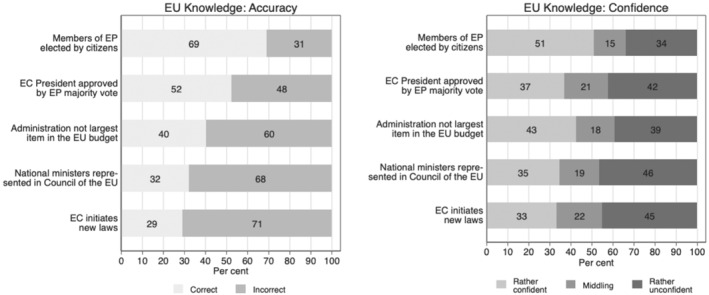
Accuracy of and Confidence in EU Knowledge *Note*: Pooled data for Austria, Denmark, France, Germany, Hungary, Italy, Poland and Spain (*N* = 16.523, weighted).

Figure 2 shows the distributions of the responses on the knowledge questions with respondents answering some questions more correctly than others. The question on the EP members being elected by citizens was answered correctly by 69 per cent of the respondents, whereas 31 per cent answered incorrectly. A slim majority, 52 per cent, also answered the question on the approval vote for the President of the European Commission correctly. The remaining three questions were answered incorrectly by a majority of the respondents. Sixty per cent were not able to identify the statement regarding the administrative costs being the largest item of the EU budget as false. Sixty‐eight per cent could not identify the institution at the EU level in which national governments are being represented by their ministers. And, 71 per cent could not name the European Commission as the institution with the formal right to initiate laws at the EU level. These apparently were harder knowledge questions.

Despite the variation in difficulty across the items measuring the accuracy of knowledge, there are fewer differences in the level of confidence, as shown by the right‐hand panel in Figure 2. Respondents felt the most confident when answering the question on the EP, with 51 per cent indicating that they felt rather confident about their answer being correct. 3 For the remaining questions the level of confidence was slightly lower ranging from 33 to 43 per cent. Overall, the patterns suggest that confidence in knowledge varies less across items than accuracy.

**Figure 3:**
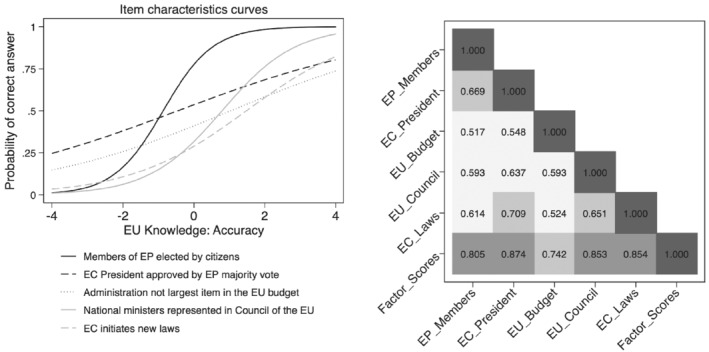
Item Characteristics Curves and Correlation Matrix *Note*: Pooled data for Austria, Denmark, France, Germany, Hungary, Italy, Poland and Spain (*N* = 16.523).

To build a scale of the accuracy of EU knowledge, we fit a two‐parameter logistic IRT model using the binary knowledge items (Thissen and Orlando, 2001). The two‐parameter model allows for varying difficulty and discrimination of the items, and is commonly identified by assuming that the latent knowledge variable follows a standard normal distribution (Bock and Lieberman, 1970). The item characteristic curves in Figure 3 confirm that the item on the EP was a fairly easy one, whereas the item of the Commission's right to initiate new laws was the hardest. At the lower end of the latent scale, the probability to answer any of the items correctly is fairly low. In contrast, at higher levels of the knowledge scale, the respondents have a fairly high propensity to answer all items correctly. The items on the EP members and the Council of the EU show the steepest slopes, meaning that they possess high discriminatory value. We extract the latent scores from the response model and use them as our measure of EU knowledge accuracy in the subsequent analyses.

To build a scale of confidence in knowledge, we ran a factor analysis on the 11‐point scales capturing confidence. The results suggest that these items strongly load on one single factor. The right‐hand panel in Figure 3 shows the correlation matrix between the individual items and the extracted factor scores. We can observe that all items are strongly correlated with this scale of confidence in knowledge. The factor scores will enter the analysis as our measure of EU knowledge confidence. 4

The correlation between our scale of accuracy of and confidence in knowledge is 0.34. Thus, although there is a moderate positive association between accuracy and confidence, we can confirm in line with previous research (Lee and Matsuo, 2018) that the two distinct analytical concepts are also empirically distinguishable.

The analysis proceeds in two steps: First, we explore the univariate and bivariate distributions of the dependent and main independent variables. Then, we run a multinomial logistic regression model as our dependent variable has three (unordered) categories. As our research design is cross‐sectional, it is particularly important to include a broad array of control variables to account for potential confounders. Several sociodemographic and non‐sociodemographic factors have been used to explain why people have chosen to vote to leave or remain in the Brexit referendum. Thereby, Leave voters were predominantly found among the more economically disadvantaged working classes, the less educated, and older voters (Goodwin and Heath, 2016). Further research indicated that also the lack of political trust, the absence of a European identity, right‐wing political leanings, anti‐immigration, anti‐free trade and anti‐establishment attitudes played a role in the vote to leave (Hobolt, 2016).

We therefore include as control variables key sociodemographic factors such as age, gender, and education as well as various measures of political attitudes and political involvement. We specifically include retrospective assessments of national and personal economic conditions, political self‐placement on the left–right and the European integration dimension, attitudes towards immigration (‘immigrants take away jobs’, ‘restricted access to welfare for immigrants’) and free trade (‘free is a good vs. bad thing’), trust in national and European institutions, a measure of European identity (‘feeling close to Europe’), and satisfaction with democracy at the EU level. We also control for political interest and a measure of internal political efficacy. Political interest is measured on a four‐point scale (from 0 ‘not interested at all’ to 3 ‘very interested’). The scale of internal political efficacy was formed based on two items (‘I have a good understanding of the important political issues facing the European Union’ and ‘I consider myself well qualified to participate in EU politics’) using factor analysis. In the same way, the measure of anti‐establishment attitudes was constructed based on two items (‘Government is run by a few big interests’, ‘Government officials use power to improve people's lives’, reversed coding). We also include country‐fixed effects along with clustered standard errors.

Apart from the control variables, the models include the above‐described scales of EU knowledge accuracy and confidence as well as an interaction between these two primary independent variables. The interaction term is particularly critical as it allows testing for the distinct effect of misinformedness, when people think they do know, but in truth they do not. If misinformedness plays a role in shaping the preference to vote leave or remain, we should see that confidence in EU knowledge is related to a preference to vote to leave at low levels accuracy, whereas it is expected to be associated with voting to remain at high level of accuracy of EU knowledge.

## IV Analysis and Results

**Figure 4:**
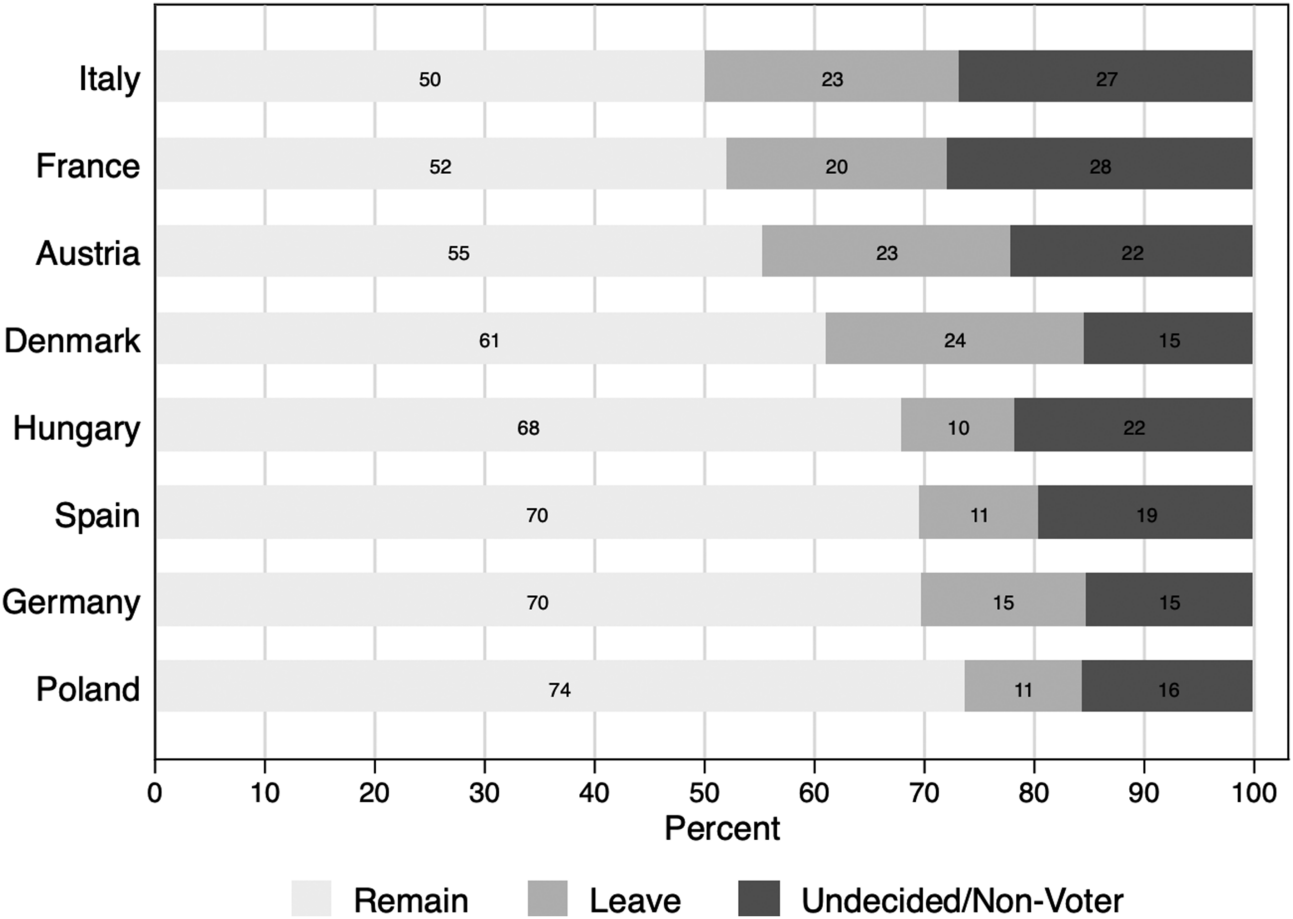
Preferences to Leave or Remain in the EU.

Figure 4 shows the distribution of preferences to remain and to leave the EU in the eight member states. In all countries under scrutiny, a majority of citizens would vote to remain in the EU. However, in some countries this majority is fairly slim, with 50 per cent in Italy, 52 per cent in France, and 55 per cent in Austria. The preference for leaving the EU is strongest in Denmark, Italy, Austria, and France, with values ranging from 20 to 24 per cent. It is smallest in the Eastern European member states, Poland and Hungary, and in Spain, with about 10 to 11 per cent. Germany, with 15 per cent of leavers, lies in the midfield. Although preferences to leave are a minority position, this minority is quite sizable in some member states. It is also worth noting that a sizable minority is undecided or preferring not to vote on this question, with the share of respondents in this group ranging from 15 to 28 per cent.

**Figure 5:**
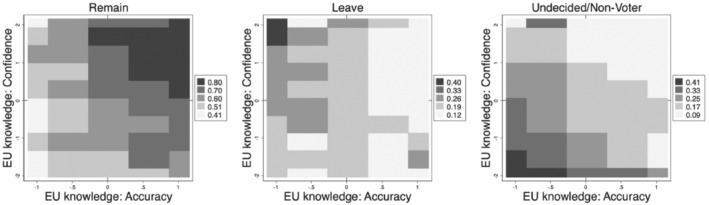
Informedness and the Preference to Leave or Remain in the EU *Note*: Pooled data for Austria, Denmark, France, Germany, Hungary, Italy, Poland and Spain (*N* = 16.523, weighted).

How are these preferences distributed across levels of EU knowledge accuracy and confidence? Figure 5 shows the heatmap of the preference to leave and remain as well as for undecided/non‐voters along the two dimensions of EU knowledge. The first map on the left‐hand side shows the share of preferences to remain. The highest shares can be found in the upper right quadrant (dark grey and black) where 73 to 81 per cent (and more) would vote to remain in a hypothetical referendum. This quadrant includes the well‐informed voters with above average accuracy (*x*‐axis) and confidence in their EU knowledge (*y*‐axis).

The second heatmap in the middle of the plot shows the distribution of preferences to leave. The maximum support is found in the most up left corner, with more than 40 per cent showing a preference to leave. But also slightly below that, at low levels of accuracy with fairly high levels of confidence, the support for leave is fairly strong. Leave support is lower at the highest levels of accurate information, in particular, among those who feel confident in their knowledge about the EU.

Finally, the third heatmap shows the distribution of the undecided and non‐voters. The highest share of undecided and non‐voters can be found in the lower left quadrant. But also in the lower right quadrant some undecided/non‐voting citizens can be found. The minimum values for undecidedness/non‐voting can be found in the upper right quadrant, hence, among the well‐informed. Overall, these descriptive results give a first support for our three hypotheses, but we now move to the multivariate analysis.

**Figure 6:**
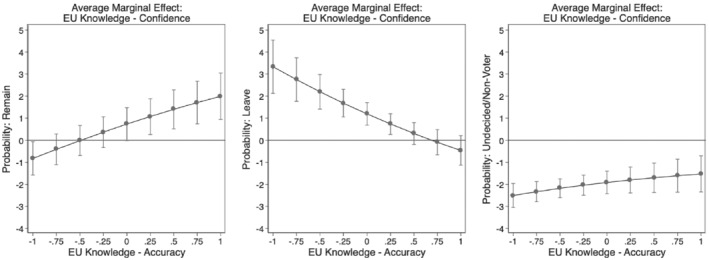
Average Marginal Effects of EU Knowledge Confidence *Note*: The dots show the average marginal effects of confidence in knowledge across varying levels of accuracy with 95% confidence intervals.

**Figure 7:**
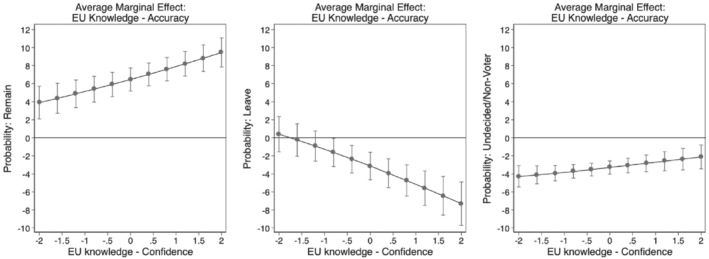
Average Marginal Effects of EU Knowledge Accuracy *Note*: The dots show the average marginal effects of accuracy in knowledge across varying levels of confidence with 95% confidence intervals.

We conduct a multinomial regression analysis of EU membership preferences, including our measures of accuracy and confidence as well as an interaction term among them alongside with our control variables. For the full estimation table, see Table B1 in Appendix B. As interaction effects are notoriously difficult to interpret in non‐linear models, we opt for a visual inspection of the continuous‐by‐continuous interaction relying on average marginal effects calculated based on the observed value approach (Hanmer and Kalkan, 2013). Specifically, we plot the average marginal effect of confidence over the range of accuracy (Figure 6) as well as the average marginal effect of accuracy over varying levels of confidence (Figure 7). 5 If our three hypotheses are confirmed, we should see in Figure 6 that confidence at high levels of accuracy is associated with a higher probability to prefer to remain (Hypothesis 1), that confidence at low levels of accuracy is associated with a higher probability to prefer to leave (Hypothesis 2), and that confidence should coincide with greater chances of being undecided/nonvoting, at all levels of accuracy (Hypothesis 3). Likewise, Figure 7 should show that accuracy at high levels of confidence relates to a greater probability of a preference to remain and to a lower chance to vote to leave.

Figures 6 and 7 confirm all three of these expectations. The first panel of Figure 6 shows the marginal effect of confidence across varying levels of accuracy on the preference to remain. In line with Hypothesis 1, we see that at the highest levels of accuracy, a unit shift in confidence is significantly associated with a higher probability of approximately 2 percentage points to vote to remain. In other words, well‐informed citizens who have a very accurate understanding of the EU and great confidence in their judgment are more likely to vote to remain in a hypothetical referendum. For the preference to leave, in line with Hypothesis 2, we see a reversed pattern in the second panel of Figure 6. At the lowest level of accuracy, a unit shift in confidence is significantly associated with a higher probability of voting to leave, of about 3.5 percentage points. In other words, misinformed voters who hold highly inaccurate EU views but who strongly feel confident about their (inaccurate) EU knowledge, are more likely to vote leave. Finally, confirming Hypothesis 3, we see in the right panel of Figure 6 a constant negative association of a unit shift in confidence on the chances of being undecided/non‐voting, with no significant variation across levels of EU knowledge accuracy. This suggests that voters who fundamentally lack confidence in how much or how little they know about the EU are most likely to remain undecided or to not vote at all – independent of their knowledge accuracy. Lastly, Figure 7 adds further confirmation to Hypotheses 1 and 2 showing that at high levels of confidence accuracy is with a higher probability to prefer remain and a lower probability of voting to leave. 6

The full results for our control variables, we document in Appendix B and only briefly summarise the main observed patterns here. We find that male respondents are more opinionated, preferring more often to vote to remain as well as to leave than female respondents, who are more likely to remain undecided or not vote at all. In line with previous research, we find that the highly educated are more likely to vote to remain and less likely to vote to leave. Interestingly, we see no significant associations with the assessment of national and personal economic conditions. In terms of political attitudes, we find that left‐wing voters prefer to vote to remain whereas right‐wing voters show a preference for leaving. Without great surprise, respondents in favour of further EU integration prefer remaining and disfavour leaving. Anti‐immigration attitudes and, most notably, perceived labour market competition (‘immigrants take away jobs’) are associated with a greater preference to leave and lower chance to vote remain. A pro‐free‐trade attitude is correlated with a higher chance to prefer remaining in the EU. When it comes to trust, we see that respondents trusting the EP show a greater chance to vote to remain rather than to leave. The same pattern emerges for a European identity and satisfaction with EU democracy: Those feeling close to Europe and those satisfied with EU democracy are more likely to vote to remain and less likely to vote to leave. In contrast, citizens with an anti‐establishment attitude, prefer to vote to leave. Concerning political involvement, we see that politically interested respondents are less likely to be undecided or not voting. For internal political efficacy – the general subjective feeling of competence in EU matters – we find that voters feeling more competent are more likely to vote to leave.

## V Robustness Checks and Discussion

The analysis thus far shows that the misinformed are more likely to prefer voting to leave the EU whereas well‐informed citizens prefer to remain. Is this a methodological artefact based on a biased selection of knowledge questions? What are the underlying causal mechanisms?

To investigate whether our knowledge items offer a particularly beneficial portrayal of the EU, we conduct two robustness checks. First, we drop two of the items that may seem to shed a somewhat beneficial light on the EU, namely the item on the approval vote for the EC president and the share of administrative costs in the budget. We rerun all steps of the analysis excluding those two items to see whether the inclusion of the specific items produced our results. This, however, is not the case as all substantial results remain completely unchanged (see Appendix C1).

Second, to assess whether misinformed voters selectively guessed more negative answers, we compare the patterns of the responses for each item between uninformed and misinformed respondents. We find that the response patterns for uninformed and misinformed voters to be fairly similar (see Appendix C2). If anything, misinformed voters selected answers that we would expect to shed a more positive light on the EU. For instance, misinformed voters more often believed that the right to propose new laws resides with the EP than uninformed or well‐informed voters. Based on these robustness checks, we conclude that our findings are not driven by single items or a particularly beneficial portrayal of EU politics. 7

The statistical association we find can be the result of two different underlying causal processes based on the theory of motivated reasoning (Lodge and Taber, 2013; Druckman and McGrath, 2019): (1) Accuracy‐motivated citizens may try to seek out objective information from sources that they subjectively believe to be highly credible and update their preferences accordingly, or (2) directionally‐motivated citizens may deliberately seek out and selectively absorb information which confirms their prior beliefs. While the goal of accuracy‐motivated reasoners is to arrive at accurate conclusions, the directionally‐motivated reasoners instead are driven by some directional goal such as maintaining their identity or affinity to a group status. Unfortunately, as Druckman and McGrath (2019) point out, these two processes are often observationally equivalent, and even cleverly designed experimental designs have struggled to disentangle those mechanisms. Previous research in general seems to suggest that the relationship between attitudes and knowledge is a mutual one (Kim *et al*., 2010). Therefore, we wish to acknowledge that reverse causality may have produced or contributed to the patterns observed.

We believe that our analysis provides a number of relevant insights and theoretical and practical conclusions that can be drawn based on the observed patterns. First, the analysis confirms that both of the two dimensions of knowledge matter and are related to preference formation in distinct ways, with the lack of confidence being associated with less opinionation and willingness to participate. Second, simply providing the misinformed with correct information may not change their views, giving their high levels of confidence in inaccurate information regarding the EU. Likewise, a part of the electorate might be quite resilient against the spread of misinformation online or elsewhere as a non‐negligible proportion of citizens already hold accurate information confidently. Finally, given that we control for various confounders, we can establish that our measurement of knowledge does not simply capture underlying motivations that have already been demonstrated to be associated with preferences regarding EU membership, but we rather add one factor relevant to our understanding of the support and opposition to the EU.

Having said that, it should also be stated that the magnitude of the association with the two dimensions of knowledge are relatively modest. Hence, if voters were to become more misinformed, it would most likely only make a substantial difference if the vote margin in a referendum would be close, which currently is not the case in the post‐Brexit EU member states. At the same time, concerns about misinformed voters should not be underestimated either, given the potentially far‐reaching consequences and as, most notably, we already know that referendum preferences can develop in highly dynamic ways, once an actual campaign is underway (LeDuc, 2009). As soon as this happens, it might be too late to build resilience against false information as this may require long‐term efforts of educating citizens about EU politics.

## Conclusion

In recent years, the concern about voters being misinformed about EU politics has been growing. The shock of the Brexit referendum has caused fears about similar events happening elsewhere and led many to question whether people understand the EU properly. While previous research has often pointed to voters being largely uninformed as a problem for democracy, voters being misinformed pose an even bigger threat to democracy. As discussed in this paper, there are good reasons to suspect that EU referendums are particularly likely to be affected by the consequences of voters being misinformed.

Based on a two‐dimensional conception of knowledge, we review the literature and identify several strands of research that link the two dimensions of knowledge with EU membership preferences. We expect misinformed voters to prefer voting to leave and well‐informed voters to prefer to vote remain. The uninformed and partially informed voters, in contrast, we expect to remain undecided or not to vote. We evaluate these expectations by making use of an original dataset covering eight EU member states and including a newly designed battery to measure different types of informedness.

The results confirm our theoretical expectations. Even when controlling for various other factors influencing preferences to leave or to remain in the EU, citizens who hold inaccurate views about the EU with great confidence are more likely to prefer voting to leave. In contrast, voters holding accurate beliefs about EU democracy with great confidence prefer voting to remain. Uninformed and partially informed voters, those with a lack of confidence in their knowledge about the EU, in general are less likely to express a clear preference. They mostly are undecided or prefer not to vote.

The fact that the preference of voting to leave is associated with citizens being misinformed has important implications for understanding the opposition to the EU.

Most notably, debunking inaccurate information about the EU by fact‐checking and educating citizens as an attempt to increase EU support among the misinformed might be deemed to fail given the high level of confidence in this group. Classical approaches such as information campaigns and fact‐checking could still, though, be effective for the uninformed and partially informed to prevent them from drifting into the group of the misinformed. As overall this group is considerably large, this is an important task for the future efforts.

Future research should focus, in particular, on examining the sources of confidence and lack of accuracy and the design of effective counterstrategies, taking into account the underlying cognitive structure of the misinformed mind. Additionally, it should also look into who is misinformed and who is uninformed about the EU, how these distributions evolve over time, and also explore the linkage between factual knowledge about the EU and systemic knowledge about political affairs.

Eventually, future research should also try to unpack the complex causal relationship between accuracy of and confidence in political knowledge on the one hand, and EU membership preferences on the other. Such tests will require longitudinal data to assess the extent to which one influences the other. This paper shows indeed that the association between accuracy of, confidence in knowledge and political decisions is indeed noteworthy and relevant to further research.

## Funding

Data collection of the RECONNECT dataset was supported by the Horizon 2020 Framework Programme (Grant no. 770142) and data collection of the AUTNES dataset was supported by the Austrian Science Fund (Grant no. S10902‐G11).

## Supporting information


**Table S1.** **B1**: Multinomial logistic regression for EU referendum preference.
**Figure S1. B1**: Average marginal effects for control variables.
**Figure S2. C1–1:** Item characteristics curves and correlation matrix (reduced scale).
**Figure S3. C1–2:** Informedness and the preference to leave or remain in the EU (reduced scale).
**Figure S4. C1–3:** Average marginal effects of EU knowledge confidence (reduced scale).
**Figure S5. C1–4:** Average marginal effects of EU knowledge accuracy (reduced scale).
**Table S6. C1–1**: Multinomial logistic regression for EU membership preference (reduced scale).
**Figure S7. C1–5**: Average marginal effects for control variables (reduced scale).
**Table S8. C2–1:** Shares of different types of informedness.
**Table S9. C2–2:** Response patterns across types of informedness.
**Figure S10. C3–1**: Item characteristics curves ‐ by country.
**Figure S11. C3–2**: Correlation matrices ‐ by country.
**Figure S12. C3–3**: Heatmaps ‐ by country.
**Figure S13. C3–4**: Average marginal effects of EU knowledge confidence ‐ by country.
**Figure S14. C3–5**: Average marginal effects of EU knowledge accuracy ‐ by country.
**Figure S15. D1**: Univariate distribution of predicted scores for accuracy and confidence.
**Figure S16. D2**: Scree plot and correlation matrix.
**Table S17**. D1. Eigenvalues.
**Table S18**. D2. Factor loadings and unique variances.
**Figure S19. E1.** Predicted probabilities for accuracy at low and high levels of confidence.
**Figure S20. E2.** Predicted probabilities for confidence at low and high levels of accuracy.

## Footnotes

^1^
It is also worth noting that our survey took place more than a month before the EP elections 2019 and hence well before the various pro‐ and anti‐EU messages could become part of the EP election campaign, which in the countries under scrutiny in this paper only started a few weeks before the elections (see Kritzinger *et al*., 2020). In the countries we cover, even during the election campaign parties' pro‐ and anti‐EU positions never really translated into a call for an EU‐exit referendum since a majority of the population was clearly against it (see Kritzinger *et al*., 2020).

^2^
Kuklinski *et al*. (2000) suggest that biased responses in their answer are suggestive of a certain direction (pro /anti‐welfare). In our case, we believe that being wrong (regardless of the direction) is most likely to lower support in the EU as it shows a lack of understanding of how the EU works. Even beliefs that suggest that the EU would be more democratic than it actually is, can lead to dissatisfaction by raising false expectations about what EU institutions can do. We believe that support for the EU requires a realistic perception of the EU to understand not only its legitimacy but also its constraints.

^3^
For visual representation the categories of the 11‐point scale of confidence in knowledge were recoded as follows: 0–4 ‘rather unconfident’, 5 ‘middling’ 6–10 ‘rather confident’.

^4^

Appendix D shows the univariate distribution of the measures of accuracy and confidence and provides additional methodological details.

^5^

Appendix E includes an alternative way of representing the continuous‐by‐continuous interaction based on predicted probabilities.

^6^
We did not derive any expectation with regard to the effect of accuracy on the chances of being undecided or a non‐voter. Yet, for completeness we also report in the third panel of Figure 7 the corresponding result. We see that accuracy at all levels of confidence decreases the chances of being undecided or non‐voting.

^7^
We have re‐run all our descriptive and multivariate analyses also country‐by‐country. The results can be found in Appendix C3. No substantial country differences could be spotted, providing evidence for a consistent association between the type of informedness and support for EU membership across countries.
