# Supplementary material for: Misinformedness about the European Union and the Preference to Vote to Leave or Remain
Source: J Common Mark Stud. 2022 Jan 24;60(5):1449–69. doi: 10.1111/jcms.13316 (PMC9544071; doi:10.1111/jcms.13316)
Supplement: Supplementary file 1 — Table S1. B1: Multinomial logistic regression for EU referendum preference. Figure S1. B1: Average marginal effects for control variables. Figure S2. C1–1: Item characteristics curves and correlation matrix (reduced scale). Figure S3. C1–2: Informedness and the preference to leave or remain in the EU (reduced scale). Figure S4. C1–3: Average marginal effects of EU knowledge confidence (reduced scale). Figure S5. C1–4: Average marginal effects of EU knowledge accuracy (reduced scale). Table S6. C1–1: Multinomial logistic regression for EU membership preference (reduced scale). Figure S7. C1–5: Average marginal effects for control variables (reduced scale). Table S8. C2–1: Shares of different types of informedness. Table S9. C2–2: Response patterns across types of informedness. Figure S10. C3–1: Item characteristics curves ‐ by country. Figure S11. C3–2: Correlation matrices ‐ by country. Figure S12. C3–3: Heatmaps ‐ by country. Figure S13. C3–4: Average marginal effects of EU knowledge confidence ‐ by country. Figure S14. C3–5: Average marginal effects of EU knowledge accuracy ‐ by country. Figure S15. D1: Univariate distribution of predicted scores for accuracy and confidence. Figure S16. D2: Scree plot and correlation matrix. Table S17. D1. Eigenvalues. Table S18. D2. Factor loadings and unique variances. Figure S19. E1. Predicted probabilities for accuracy at low and high levels of confidence. Figure S20. E2. Predicted probabilities for confidence at low and high levels of accuracy. [file JCMS-60-1449-s001.docx]

**SUPPLEMENTARY MATERIAL**

**ONLINE-ONLY APPENDIX**

**Appendix A: Question wording**

**Appendix B: Estimation results**

**Appendix C: Robustness checks**

**Appendix D: Details on measurement scales**

**Appendix E: Alternative representation of model predictions**

**Appendix A: Question wording**

**A1. Questions to measure the accuracy of knowledge**

The order of the questions was randomised. Correct answers marked with an asterisk. Response options were all randomised where appropriate (exception: “None/all of the above).

The members of which of the following EU institutions are directly elected by European citizens?

- The European Commission
- The European Parliament*
- The Council of the European Union
- None of the above

Jean-Claude Juncker is the current President of the European Commission. How did he become President?

- He was directly elected by EU citizens.
- He was selected on the basis of an internal vote within the European Commission.
- He was approved by a majority of the members of the European Parliament.*
- He was appointed without a vote taking place.

Which of the following EU institutions formally proposes new laws at the EU level?

- The European Commission*
- The European Parliament
- The Council of the European Union
- All of the above

Which of the following statements is not true?

- All EU citizens have the right to work in another EU country.
- Mobile phone roaming charges have decreased within the EU.
- Expenditure on the salaries of the EU officials is the largest item in the EU budget.*
- Individual EU countries cannot conclude their own trade agreements.

In which of the following EU institutions are the ministers of national governments represented?

- The European Commission
- The European Parliament
- The Council of the European Union*
- None of the above

**A2. Questions to measure the confidence in knowledge**

Each question above was followed by the following question.

How sure are you about the answer you have just given?

- 0 = Not at all sure
- …
- 10 = Completely sure

**Appendix B: Estimation results**

**Table B1**: Multinomial logistic regression for EU referendum preference

|  | (1) | | | | (2) | | | |
| --- | --- | --- | --- | --- | --- | --- | --- | --- |
|  | Baseline Model | | | | Full Model | | | |
|  | Remain | | Leave | | Remain | | Leave | |
| EU Knowledge: Accuracy |  |  |  |  | 0.473^***^ | (0.039) | -0.096 | (0.080) |
| EU Knowledge: Confidence |  |  |  |  | 0.133^***^ | (0.029) | 0.206^***^ | (0.026) |
| EU Knowledge: Accuracy X Confidence |  |  |  |  | 0.060^**^ | (0.020) | -0.154^***^ | (0.043) |
| Age (ref. <30 years): |  |  |  |  |  |  |  |  |
| - 30-39 years | -0.169^*^ | (0.082) | -0.041 | (0.088) | -0.174^*^ | (0.081) | -0.042 | (0.091) |
| - 40-49 years | -0.102 | (0.089) | -0.133 | (0.122) | -0.154 | (0.092) | -0.151 | (0.124) |
| - 50-59 years | 0.012 | (0.059) | -0.056 | (0.123) | -0.094 | (0.069) | -0.096 | (0.117) |
| - 60+ years | 0.237^*^ | (0.105) | -0.264^*^ | (0.105) | 0.119 | (0.097) | -0.329^***^ | (0.098) |
| Gender: Male | 0.425^***^ | (0.105) | 0.294^***^ | (0.082) | 0.288^**^ | (0.095) | 0.209^*^ | (0.083) |
| Education (ref. Low): |  |  |  |  |  |  |  |  |
| - Medium | 0.235^***^ | (0.053) | -0.188 | (0.112) | 0.199^***^ | (0.048) | -0.190 | (0.115) |
| - High | 0.530^***^ | (0.069) | -0.292^*^ | (0.121) | 0.414^***^ | (0.068) | -0.315^**^ | (0.122) |
| Political Interest | 0.203^***^ | (0.044) | 0.309^***^ | (0.024) | 0.125^**^ | (0.040) | 0.248^***^ | (0.025) |
| Internal Efficacy | -0.026 | (0.033) | 0.088^***^ | (0.026) | -0.050 | (0.026) | 0.068^*^ | (0.027) |
| - Left | 0.426^**^ | (0.154) | 0.158 | (0.123) | 0.409^**^ | (0.149) | 0.182 | (0.124) |
| - Right | -0.080 | (0.095) | 0.507^***^ | (0.063) | -0.080 | (0.093) | 0.506^***^ | (0.063) |
| National Economic Conditions | 0.001 | (0.064) | -0.042 | (0.051) | 0.002 | (0.061) | -0.042 | (0.048) |
| Personal Economic Conditions | 0.069^*^ | (0.031) | 0.018 | (0.045) | 0.064 | (0.033) | 0.019 | (0.042) |
| Position: Immigrants Take Away Jobs | -0.063^***^ | (0.015) | 0.032^*^ | (0.014) | -0.055^***^ | (0.015) | 0.032^*^ | (0.014) |
| Position: Restricted Welfare For Immigrants | 0.009 | (0.007) | 0.010 | (0.007) | 0.008 | (0.007) | 0.014 | (0.007) |
| Attitude Towards Free Trade | 0.457^***^ | (0.099) | -0.134 | (0.069) | 0.443^***^ | (0.093) | -0.129 | (0.070) |
| Position: EU Integration | 0.191^***^ | (0.017) | -0.111^***^ | (0.015) | 0.194^***^ | (0.017) | -0.111^***^ | (0.015) |
| Satisfaction with Democracy in the EU | 0.114^***^ | (0.021) | -0.045^*^ | (0.021) | 0.119^***^ | (0.019) | -0.042 | (0.022) |
| Feeling close to Europe | 0.737^***^ | (0.068) | -0.376^***^ | (0.028) | 0.717^***^ | (0.065) | -0.376^***^ | (0.031) |
| Trust: European Commission | 0.041 | (0.029) | -0.000 | (0.036) | 0.054 | (0.030) | -0.008 | (0.037) |
| Trust: European Parliament | 0.119^***^ | (0.023) | -0.050^**^ | (0.018) | 0.111^***^ | (0.024) | -0.049^**^ | (0.018) |
| Trust: National Government | -0.030 | (0.034) | -0.026 | (0.029) | -0.045 | (0.033) | -0.028 | (0.029) |
| Trust: National Parliament | -0.033 | (0.032) | 0.030 | (0.023) | -0.033 | (0.032) | 0.026 | (0.021) |
| Anti-Establishment Attitude | 0.017 | (0.024) | 0.054^*^ | (0.022) | 0.011 | (0.024) | 0.051^**^ | (0.020) |
| Country (ref. Germany): |  |  |  |  |  |  |  |  |
| - France | -0.056 | (0.046) | -0.437^***^ | (0.033) | -0.026 | (0.046) | -0.411^***^ | (0.029) |
| - Spain | -0.347^***^ | (0.045) | -0.256^***^ | (0.066) | -0.371^***^ | (0.042) | -0.288^***^ | (0.066) |
| - Hungary | -0.309^**^ | (0.098) | -0.600^***^ | (0.022) | -0.394^***^ | (0.089) | -0.695^***^ | (0.019) |
| - Poland | -0.140 | (0.089) | -0.093^*^ | (0.039) | -0.203^**^ | (0.077) | -0.146^***^ | (0.041) |
| - Denmark | -0.161^***^ | (0.043) | 0.752^***^ | (0.034) | -0.175^***^ | (0.039) | 0.740^***^ | (0.033) |
| - Italy | -0.645^***^ | (0.050) | -0.267^***^ | (0.052) | -0.667^***^ | (0.046) | -0.323^***^ | (0.056) |
| - Austria | 0.033 | (0.040) | -0.065 | (0.034) | 0.026 | (0.041) | -0.075^*^ | (0.032) |
| Constant | -2.547^***^ | (0.160) | 0.299^*^ | (0.120) | -2.158^***^ | (0.171) | 0.580^***^ | (0.118) |
| Observations | 14771 | | | | 14771 | | | |
| McFadden Pseudo-*R*^2^ | 0.303 | | | | 0.312 | | | |
| *AIC* | 17697 | | | | 17471 | | | |
| *BIC* | 17751 | | | | 17524 | | | |

*Note*: The base category is “undecided/non-voter”. Entries are coefficients from multinomial logistic regression. Cluster-robust standard errors in parentheses.

^*^ *p* < 0.05, ^**^ *p* < 0.01, ^***^ *p* < 0.001.

**Figure B1**: Average marginal effects for control variables
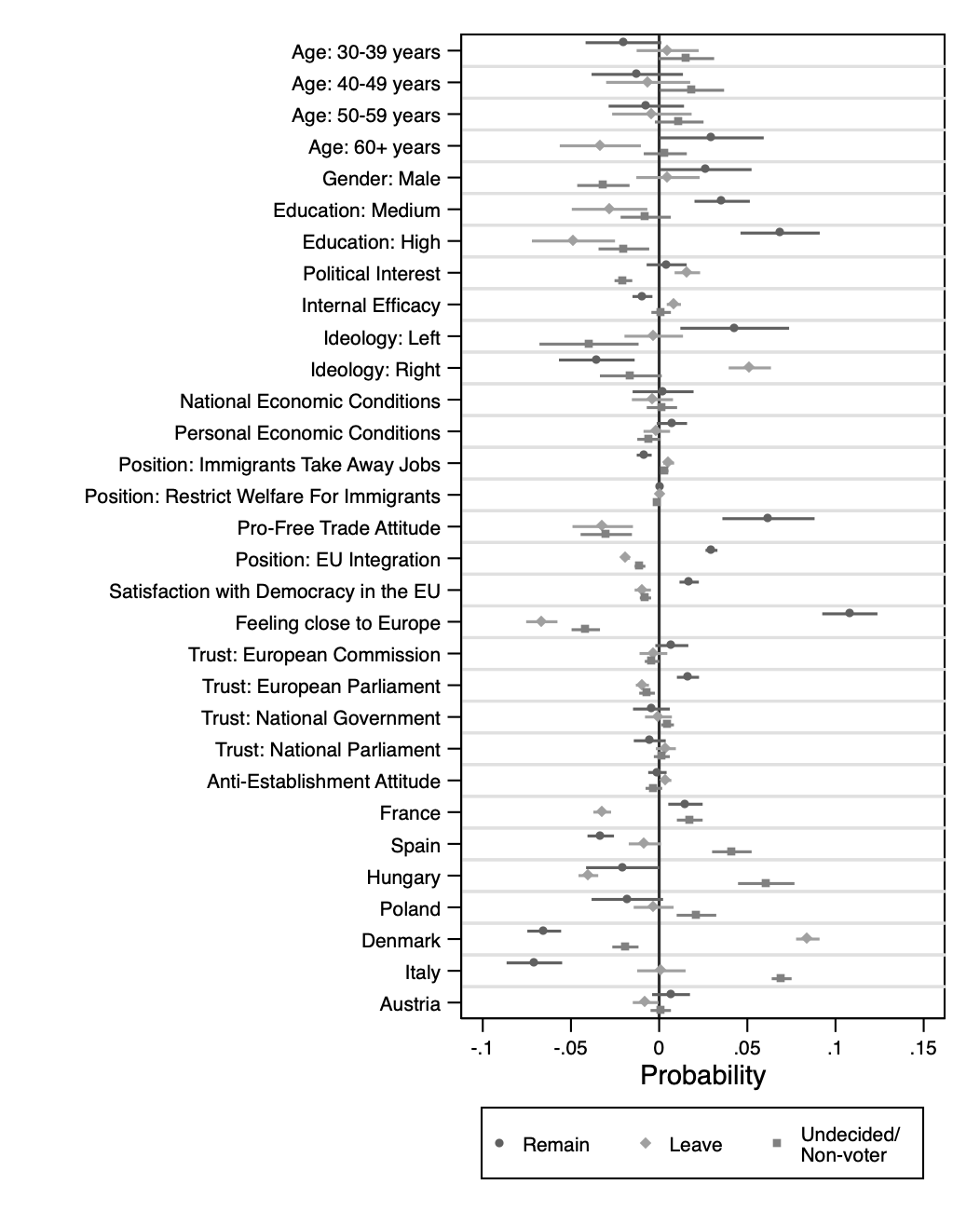


*Note*: Based on full model in Table B1.

**Appendix C: Robustness checks**

**C1. Removing items from the knowledge scale**

To evaluate whether our results are contingent on the selection of specific items, we replicate all steps of the analysis removing two of the items (EC President, EU budget). The correlation between the scale using all items with the reduced scale is 0.97*** for both confidence and accuracy. As can be seen below, all results remain unchanged. This suggests that the results are not driven by the selection of a subset of the items.

**Figure C1-1:** Item characteristics curves and correlation matrix (reduced scale)

**
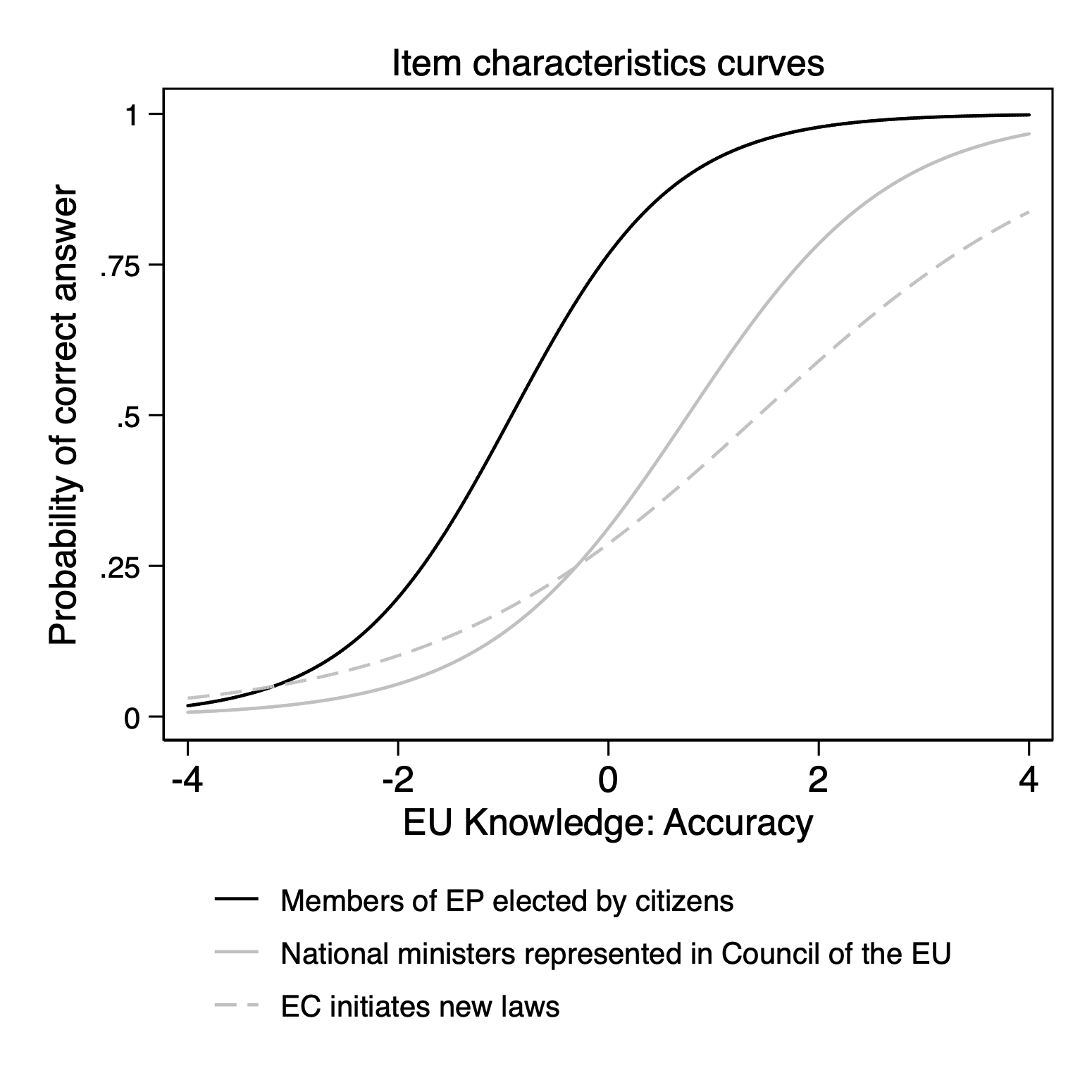

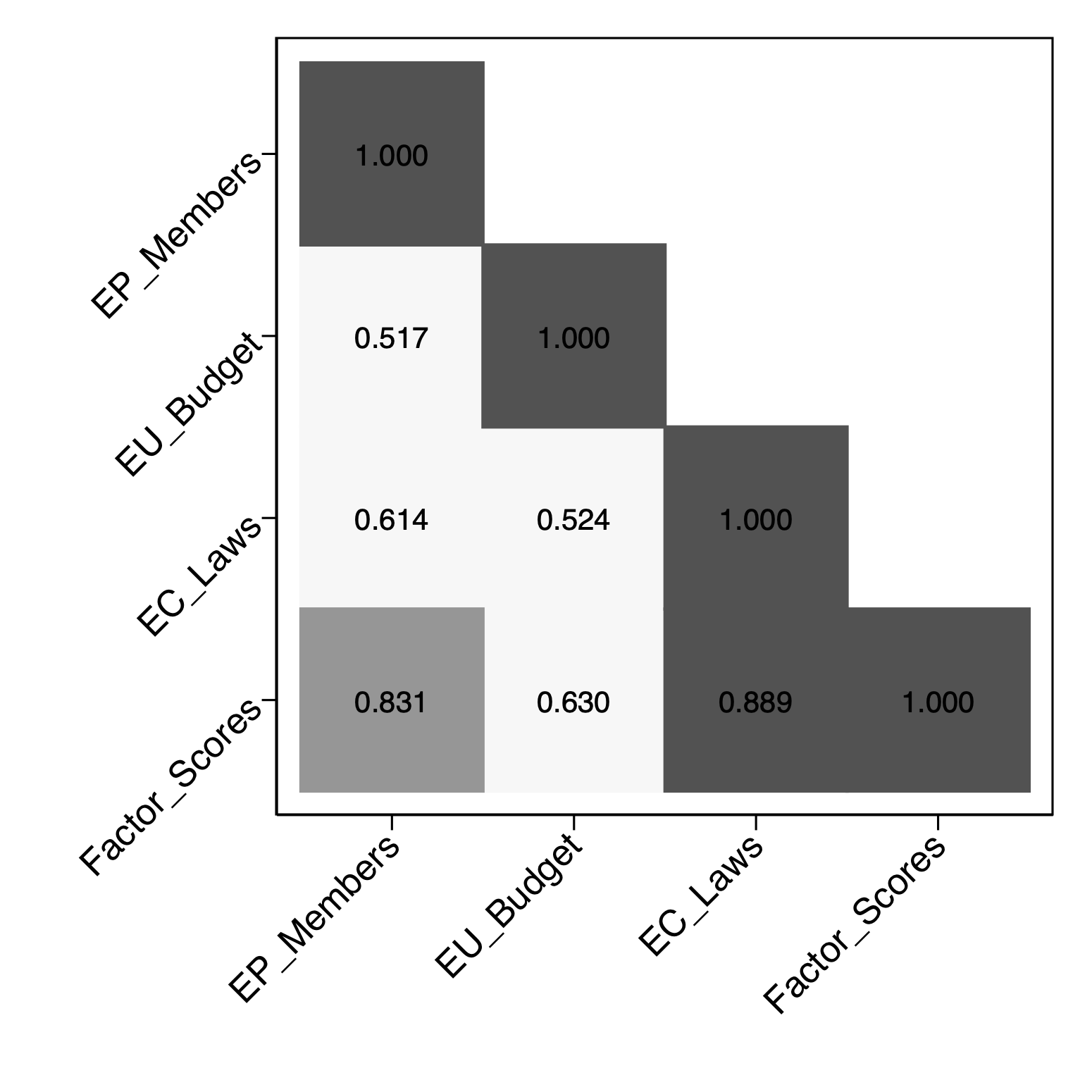
**

**Figure C1-2:** Informedness and the preference to leave or remain in the EU (reduced scale)**
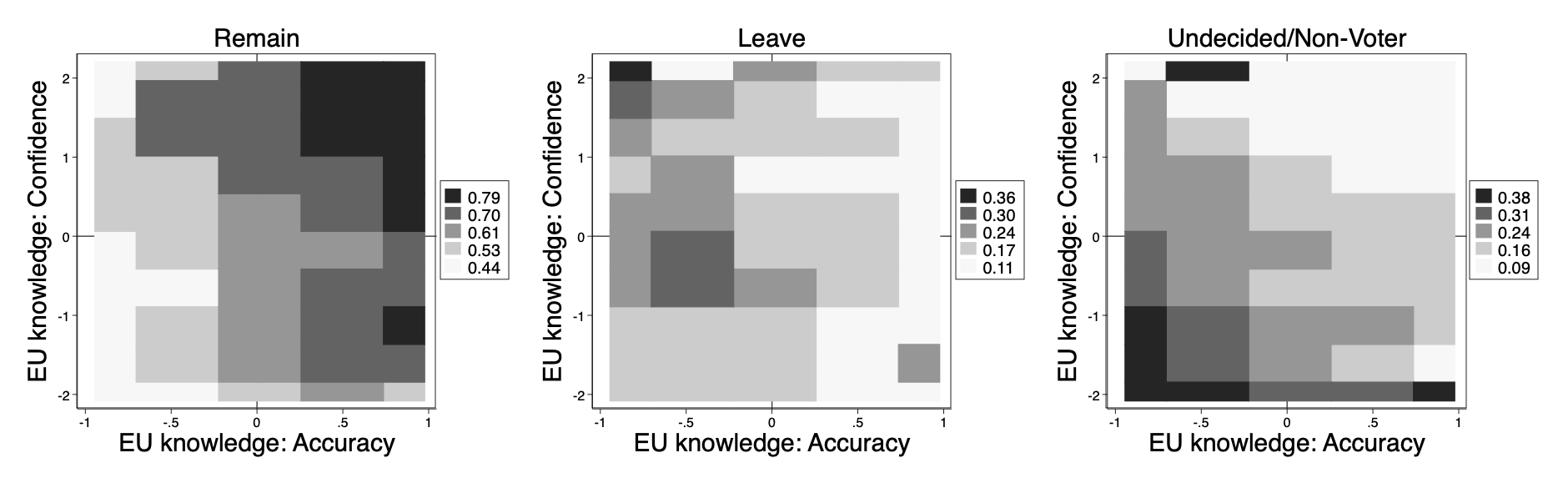
**

*Note*: Pooled data for Austria, Denmark, France, Germany, Hungary, Italy, Poland and Spain (N=16.523, weighted).

**Figure C1-3:** Average marginal effects of EU knowledge confidence (reduced scale)


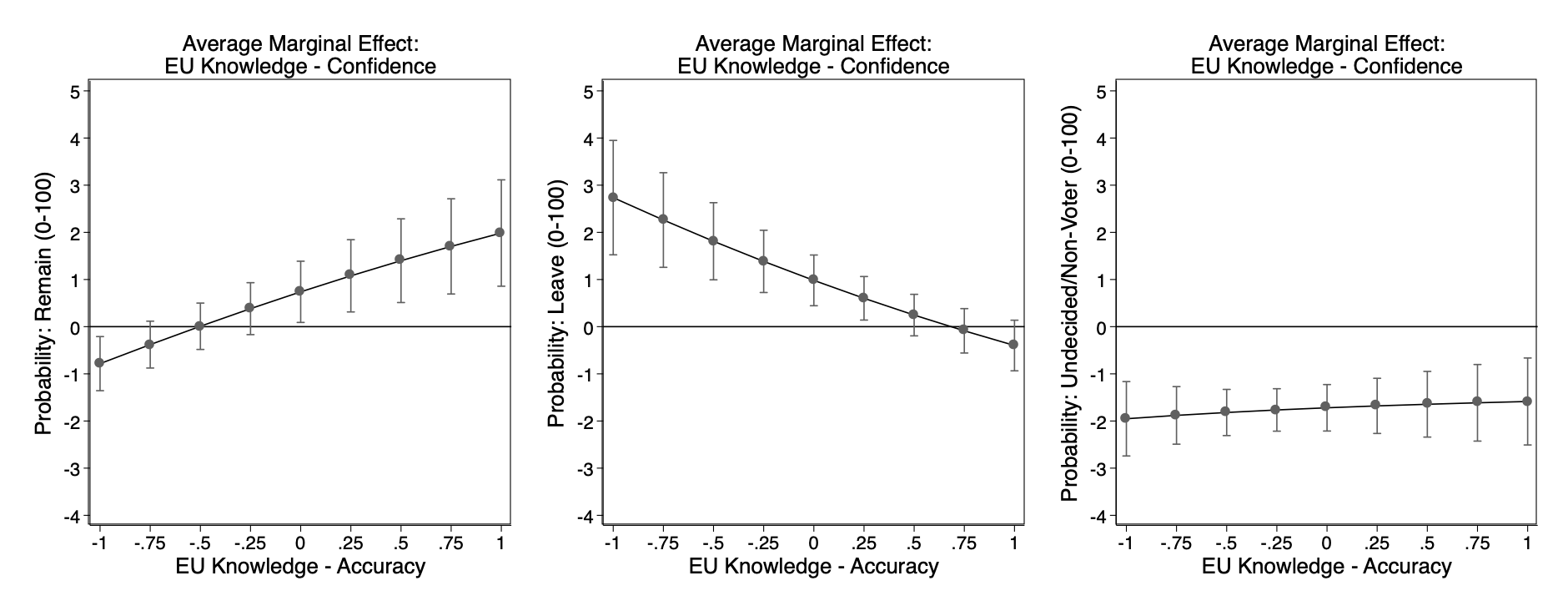


Note: The dots show the average marginal effects of confidence in knowledge across varying levels of accuracy with 95% confidence intervals.

**Figure C1-4:** Average marginal effects of EU knowledge accuracy (reduced scale)


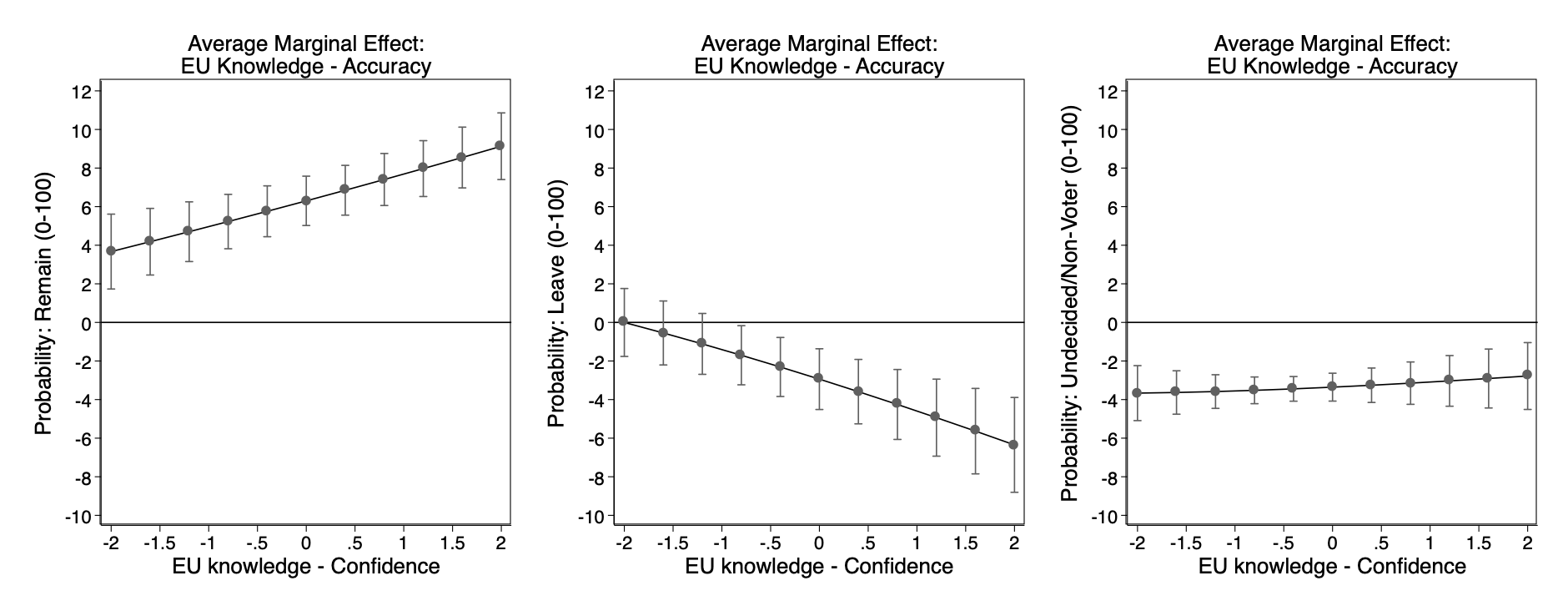


Note: The dots show the average marginal effects of confidence in knowledge across varying levels of accuracy with 95% confidence intervals.

**Table C1-1**: Multinomial logistic regression for EU membership preference (reduced scale)

|  | (1)  Baseline Model | | |  | (2)  Full Model | | | |
| --- | --- | --- | --- | --- | --- | --- | --- | --- |
|  |  |  |  |  |  |  |  |  |
|  | Remain |  | Leave |  | Remain |  | Leave |  |
| EU Knowledge: Accuracy |  |  |  |  | 0.467^***^ | (0.035) | -0.078 | (0.084) |
| EU Knowledge: Certainty |  |  |  |  | 0.123^***^ | (0.025) | 0.176^***^ | (0.030) |
| EU Knowledge: Accuracy X Scores |  |  |  |  | 0.073^*^ | (0.032) | -0.114^**^ | (0.041) |
| - 30-39 years | -0.169^*^ | (0.082) | -0.041 | (0.088) | -0.169^*^ | (0.083) | -0.034 | (0.093) |
| - 40-49 years | -0.102 | (0.089) | -0.133 | (0.122) | -0.144 | (0.093) | -0.141 | (0.123) |
| - 50-59 years | 0.012 | (0.059) | -0.056 | (0.123) | -0.078 | (0.068) | -0.086 | (0.119) |
| - 60+ years | 0.237^*^ | (0.105) | -0.264^*^ | (0.105) | 0.132 | (0.098) | -0.315^**^ | (0.100) |
| Gender: Male | 0.425^***^ | (0.105) | 0.294^***^ | (0.082) | 0.300^**^ | (0.096) | 0.223^**^ | (0.081) |
| - Medium | 0.235^***^ | (0.053) | -0.188 | (0.112) | 0.205^***^ | (0.047) | -0.187 | (0.114) |
| - High | 0.530^***^ | (0.069) | -0.292^*^ | (0.121) | 0.419^***^ | (0.069) | -0.311^**^ | (0.119) |
| Political Interest | 0.203^***^ | (0.044) | 0.309^***^ | (0.024) | 0.128^**^ | (0.039) | 0.255^***^ | (0.025) |
| Internal Efficacy | -0.026 | (0.033) | 0.088^***^ | (0.026) | -0.047 | (0.026) | 0.071^*^ | (0.028) |
| - Left | 0.426^**^ | (0.154) | 0.158 | (0.123) | 0.411^**^ | (0.150) | 0.180 | (0.125) |
| - Right | -0.080 | (0.095) | 0.507^***^ | (0.063) | -0.083 | (0.094) | 0.507^***^ | (0.062) |
| National Economic Conditions | 0.001 | (0.064) | -0.042 | (0.051) | 0.002 | (0.061) | -0.041 | (0.048) |
| Personal Economic Conditions | 0.069^*^ | (0.031) | 0.018 | (0.045) | 0.065^*^ | (0.033) | 0.016 | (0.042) |
| Position: Immigrants Take Away Jobs | -0.063^***^ | (0.015) | 0.032^*^ | (0.014) | -0.056^***^ | (0.015) | 0.033^*^ | (0.014) |
| Position: Restrict Welfare For Immigrants | 0.009 | (0.007) | 0.010 | (0.007) | 0.009 | (0.007) | 0.012 | (0.007) |
| Pro-Free Trade Attitude | 0.457^***^ | (0.099) | -0.134 | (0.069) | 0.447^***^ | (0.093) | -0.130 | (0.070) |
| Position: EU Integration | 0.191^***^ | (0.017) | -0.111^***^ | (0.015) | 0.194^***^ | (0.016) | -0.111^***^ | (0.015) |
| Satisfaction with Democracy in the EU | 0.114^***^ | (0.021) | -0.045^*^ | (0.021) | 0.120^***^ | (0.019) | -0.042 | (0.022) |
| Feeling close to Europe | 0.737^***^ | (0.068) | -0.376^***^ | (0.028) | 0.718^***^ | (0.065) | -0.377^***^ | (0.031) |
| Trust: European Commission | 0.041 | (0.029) | -0.000 | (0.036) | 0.055 | (0.030) | -0.006 | (0.037) |
| Trust: European Parliament | 0.119^***^ | (0.023) | -0.050^**^ | (0.018) | 0.111^***^ | (0.025) | -0.050^**^ | (0.019) |
| Trust: National Government | -0.030 | (0.034) | -0.026 | (0.029) | -0.044 | (0.033) | -0.029 | (0.029) |
| Trust: National Parliament | -0.033 | (0.032) | 0.030 | (0.023) | -0.034 | (0.032) | 0.028 | (0.022) |
| Anti-Establishment Attitude | 0.017 | (0.024) | 0.054^*^ | (0.022) | 0.012 | (0.024) | 0.052^*^ | (0.020) |
| - France | -0.056 | (0.046) | -0.437^***^ | (0.033) | -0.050 | (0.047) | -0.431^***^ | (0.029) |
| - Spain | -0.347^***^ | (0.045) | -0.256^***^ | (0.066) | -0.389^***^ | (0.043) | -0.300^***^ | (0.068) |
| - Hungary | -0.309^**^ | (0.098) | -0.600^***^ | (0.022) | -0.392^***^ | (0.090) | -0.701^***^ | (0.020) |
| - Poland | -0.140 | (0.089) | -0.093^*^ | (0.039) | -0.219^**^ | (0.078) | -0.152^***^ | (0.040) |
| - Denmark | -0.161^***^ | (0.043) | 0.752^***^ | (0.034) | -0.181^***^ | (0.040) | 0.725^***^ | (0.032) |
| - Italy | -0.645^***^ | (0.050) | -0.267^***^ | (0.052) | -0.685^***^ | (0.046) | -0.336^***^ | (0.059) |
| - Austria | 0.033 | (0.040) | -0.065 | (0.034) | 0.028 | (0.041) | -0.073^*^ | (0.033) |
| Constant | -2.547^***^ | (0.160) | 0.299^*^ | (0.120) | -2.188^***^ | (0.171) | 0.551^***^ | (0.117) |
| Observations | 14771 | | | | 14771 | | | |
| Pseudo *R*^2^ | 0.303 | | | | 0.311 | | | |
| *AIC* | 17697 | | | | 17499 | | | |
| *BIC* | 17751 | | | | 17552 | | | |

*Note*: The base category is “undecided/non-voter”. Entries are coefficients from multinomial logistic regression. Cluster-robust standard errors in parentheses.

^*^ *p* < 0.05, ^**^ *p* < 0.01, ^***^ *p* < 0.001.

**Figure C1-5**: Average marginal effects for control variables (reduced scale)


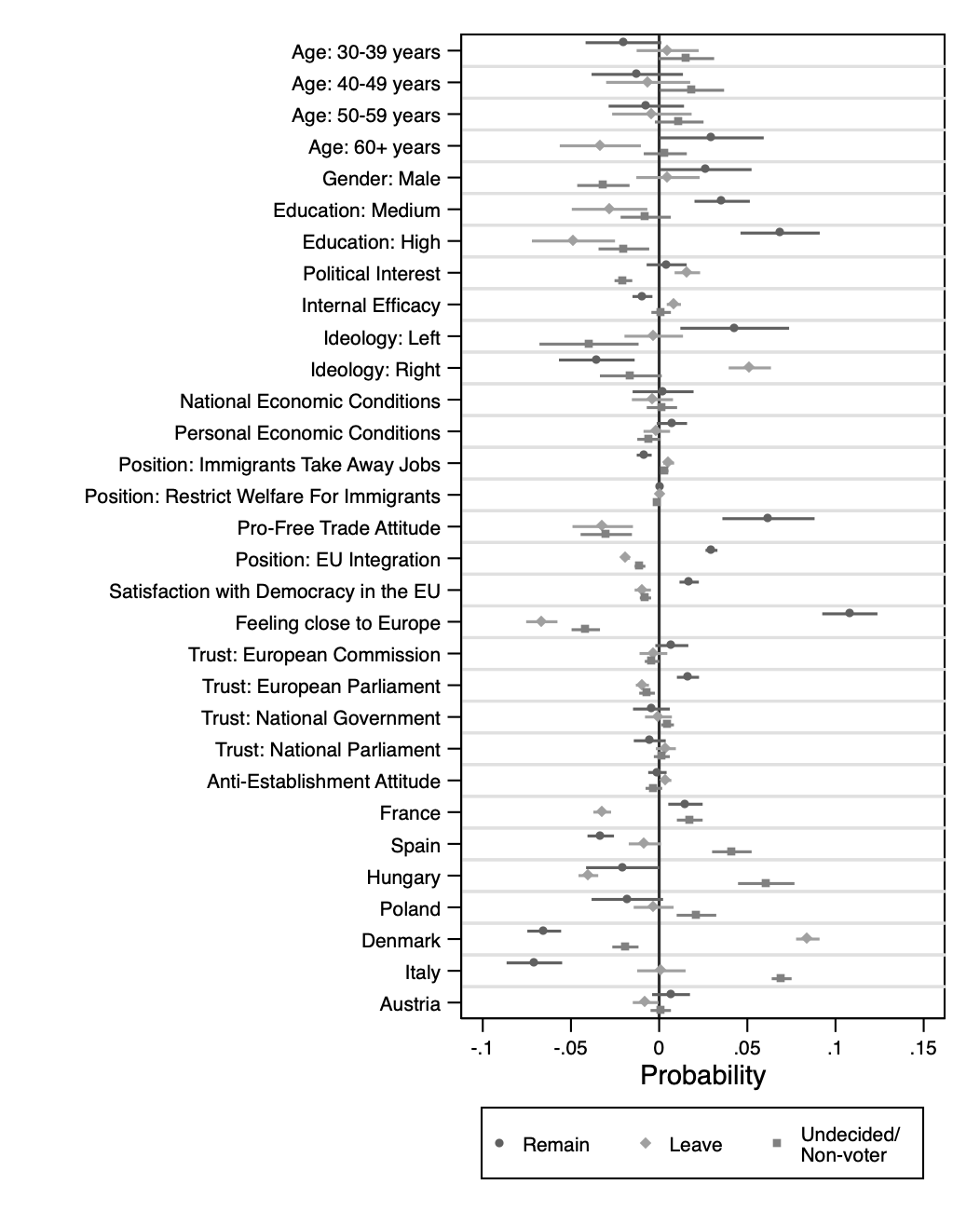


*Note*: Based on full model in Table C1-1.

**C2. Types of informedness and response patterns**

In the following, we describe the shares of different types of respondents as well as the patterns of responses among them. For this purpose, respondents were classified as (1) well-informed when their accuracy and confidence were above the mean (>0), as (2) partially informed when scores for accuracy were above (>0) but confidence below the average (≤0), as (3) misinformed when accuracy was below (≤0) but confidence above average (>0), and as uninformed when both accuracy and confidence were below average (≤0).^[[1]](#footnote-1)^ Table C2-1 reports the share for each type of informedness by country under investigation based on these coding rules. Table C2-2 shows the column percentages of each single knowledge item for each type as well as for the entire sample.

**Table C2-1:** Shares of different types of informedness

|  | Well-informed | Partially informed | Misinformed | Uninformed | Total |
| --- | --- | --- | --- | --- | --- |
| Germany | 32.1 | 18.3 | 19.3 | 30.3 | 100.0 |
| France | 22.4 | 17.4 | 18.0 | 42.2 | 100.0 |
| Spain | 28.9 | 17.8 | 21.0 | 32.3 | 100.0 |
| Hungary | 35.6 | 12.5 | 26.1 | 25.8 | 100.0 |
| Poland | 36.0 | 14.0 | 24.9 | 25.1 | 100.0 |
| Denmark | 29.9 | 16.2 | 20.9 | 33.0 | 100.0 |
| Italy | 29.6 | 12.2 | 28.3 | 29.9 | 100.0 |
| Austria | 44.9 | 12.8 | 16.8 | 25.5 | 100.0 |
| Total | 32.4 | 15.2 | 21.9 | 30.5 | 100.0 |

*Note*: Entries are row percentages. Pooled data for Austria, Denmark, France, Germany, Hungary, Italy, Poland and Spain (N=16.523); weighted.

**Table C2-2:** Response patterns across types of informedness

| The members of which of the following EU institutions are directly elected by European citizens? | Well-informed | Partially informed | Misinformed | Uninformed | Total |
| --- | --- | --- | --- | --- | --- |
| The European Commission | 0.3 | 0.9 | 15.1 | 19.5 | 9.8 |
| The European Parliament* | 98.9 | 96.1 | 56.9 | 35.6 | 69.1 |
| The Council of the European Union | 0.4 | 1.1 | 11.4 | 14.5 | 7.4 |
| None of the above | 0.4 | 1.9 | 16.6 | 30.4 | 13.7 |
| Jean-Claude Juncker is the current President of the European Commission. How did he become President? | Well-informed | Partially informed | Misinformed | Uninformed | Total |
| He was directly elected by EU citizens. | 1.4 | 3.4 | 6.0 | 8.0 | 4.8 |
| He was selected on the basis of an internal vote within the European Commission. | 28.3 | 29.9 | 43.7 | 37.6 | 34.9 |
| He was approved by a majority of the members of the European Parliament.* | 65.2 | 60.9 | 41.7 | 43.5 | 52.5 |
| He was appointed without a vote taking place. | 5.1 | 5.8 | 8.6 | 10.9 | 7.8 |
| Which of the following EU institutions formally proposes new laws at the EU level? | Well-informed | Partially informed | Misinformed | Uninformed | Total |
| The European Commission* | 51.2 | 52.7 | 8.6 | 11.0 | 29.2 |
| The European Parliament | 25.9 | 23.8 | 49.0 | 43.4 | 36.3 |
| The Council of the European Union | 10.1 | 11.1 | 21.4 | 20.1 | 15.9 |
| All of the above | 12.8 | 12.4 | 21.0 | 25.5 | 18.6 |
| Which of the following statements is not true? | Well-informed | Partially informed | Misinformed | Uninformed | Total |
| All EU citizens have the right to work in another EU country. | 7.7 | 9.5 | 15.5 | 16.1 | 12.4 |
| Mobile phone roaming charges have decreased within the EU. | 7.9 | 11.1 | 17.5 | 20.9 | 14.6 |
| Expenditure on the salaries of the EU officials is the largest item in the EU budget.* | 55.1 | 51.1 | 29.6 | 28.8 | 40.5 |
| Individual EU countries cannot conclude their own trade agreements. | 29.3 | 28.3 | 37.5 | 34.1 | 32.5 |
| In which of the following EU institutions are the ministers of national governments represented? | Well-informed | Partially informed | Misinformed | Uninformed | Total |
| The European Commission | 12.9 | 16.5 | 29.3 | 23.9 | 20.6 |
| The European Parliament | 16.9 | 23.4 | 45.0 | 38.9 | 31.2 |
| The Council of the European Union* | 63.0 | 49.9 | 8.5 | 10.8 | 32.3 |
| None of the above | 7.2 | 10.3 | 17.3 | 26.4 | 16.0 |

*Note*: Entries are column percentages. Pooled data for Austria, Denmark, France, Germany, Hungary, Italy, Poland and Spain (N=16.523).

**C3. Country-by-country analysis**

**Figure C3-1**: Item characteristics curves - by country

| Germany | France | Spain | Hungary |
| --- | --- | --- | --- |
| 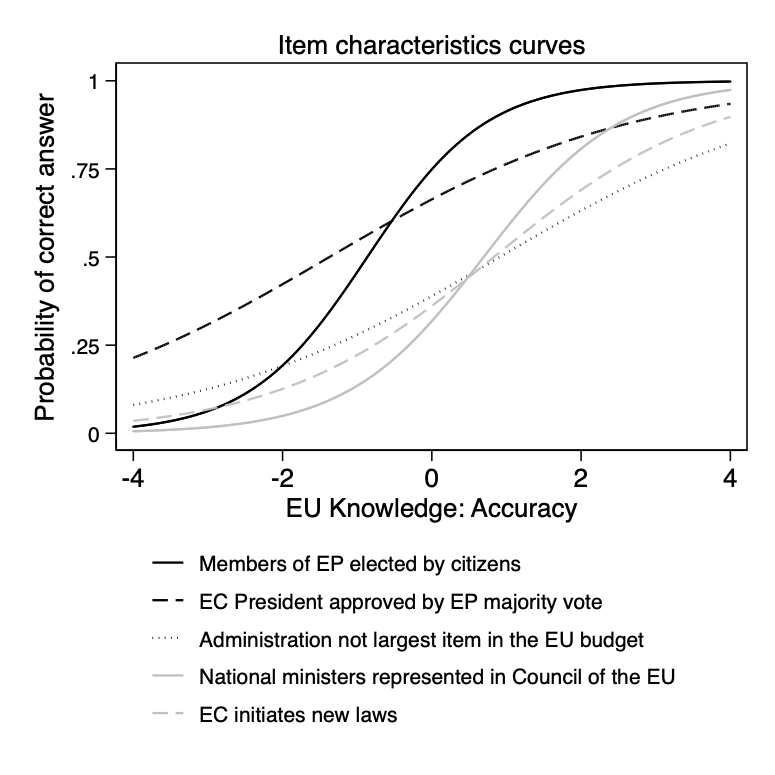 | 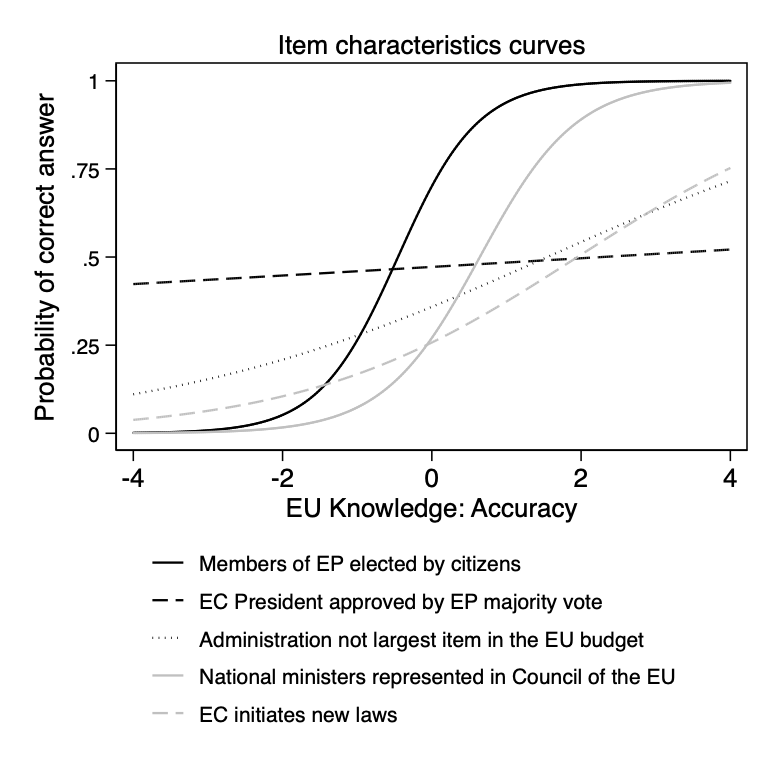 | 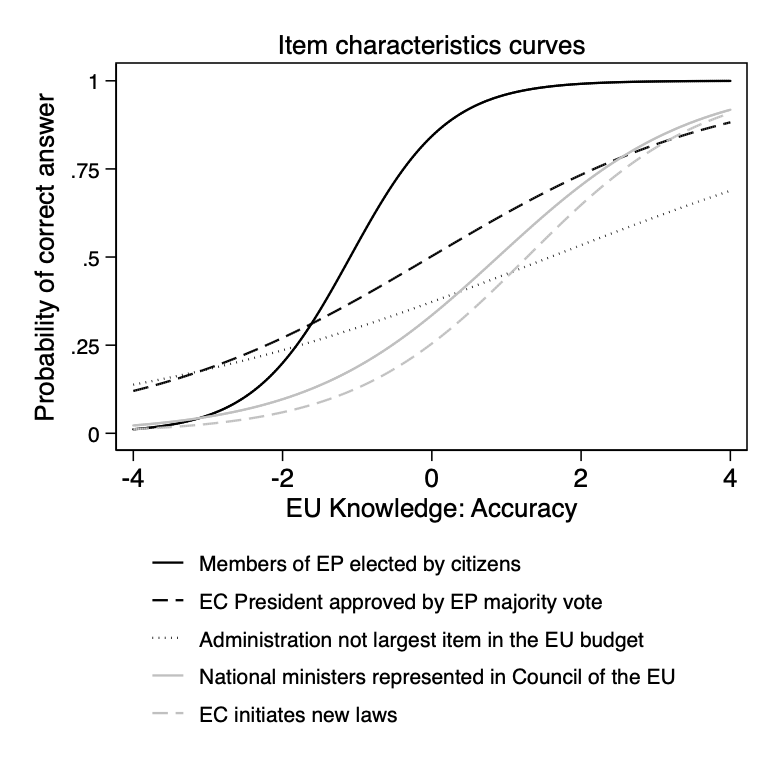 | 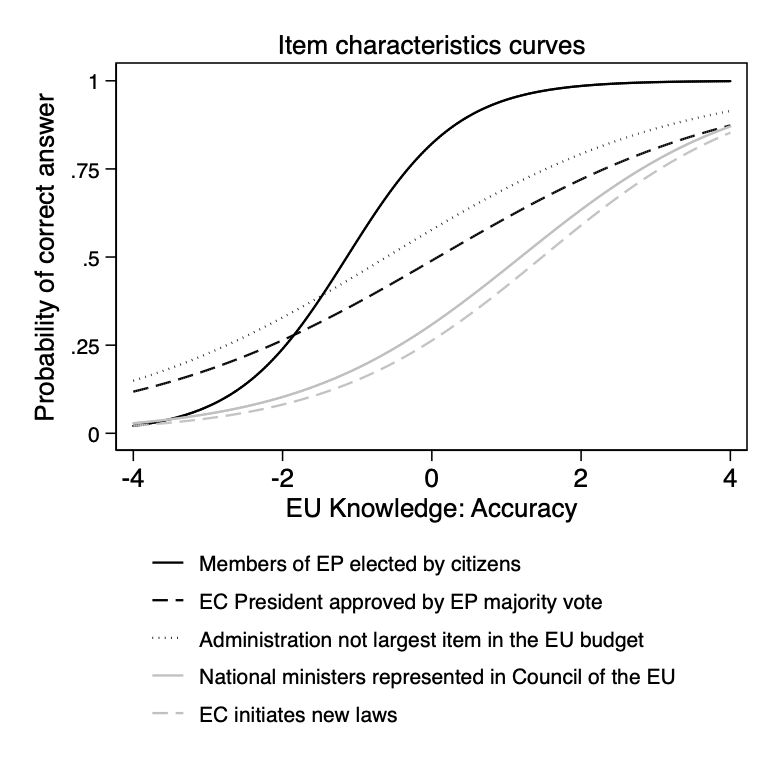 |
| Poland | Denmark | Italy | Austria |
| 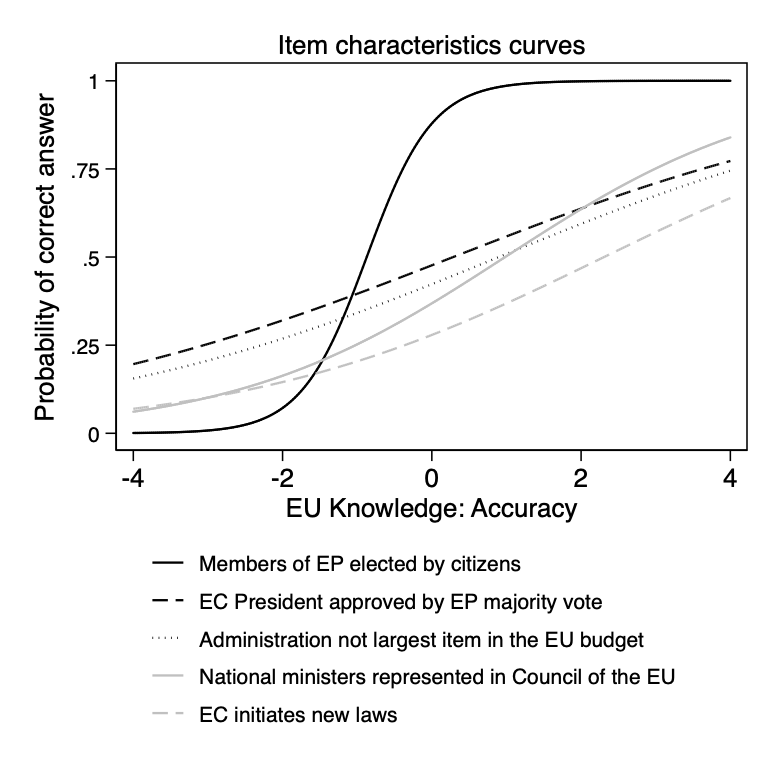 | 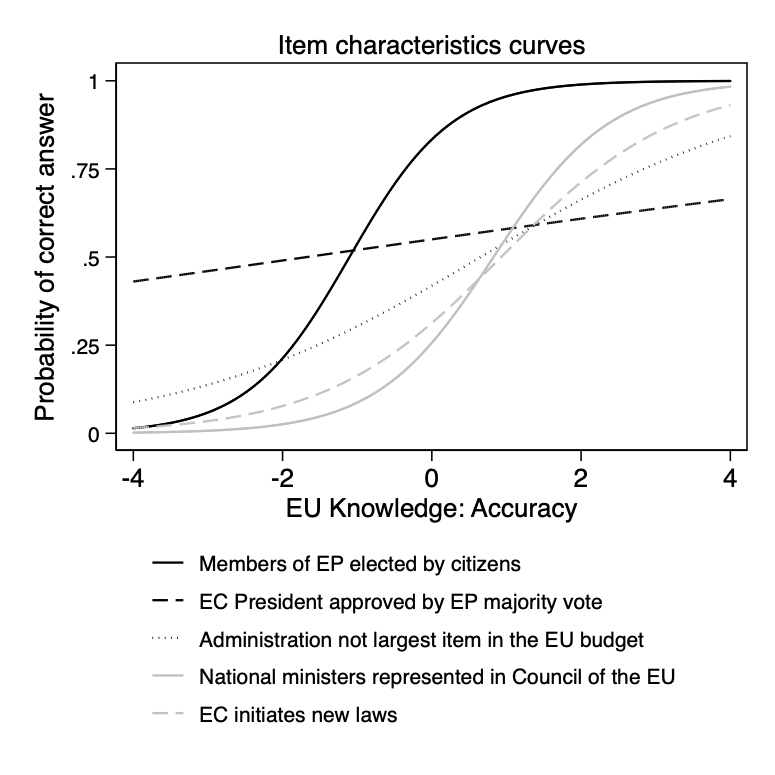 | 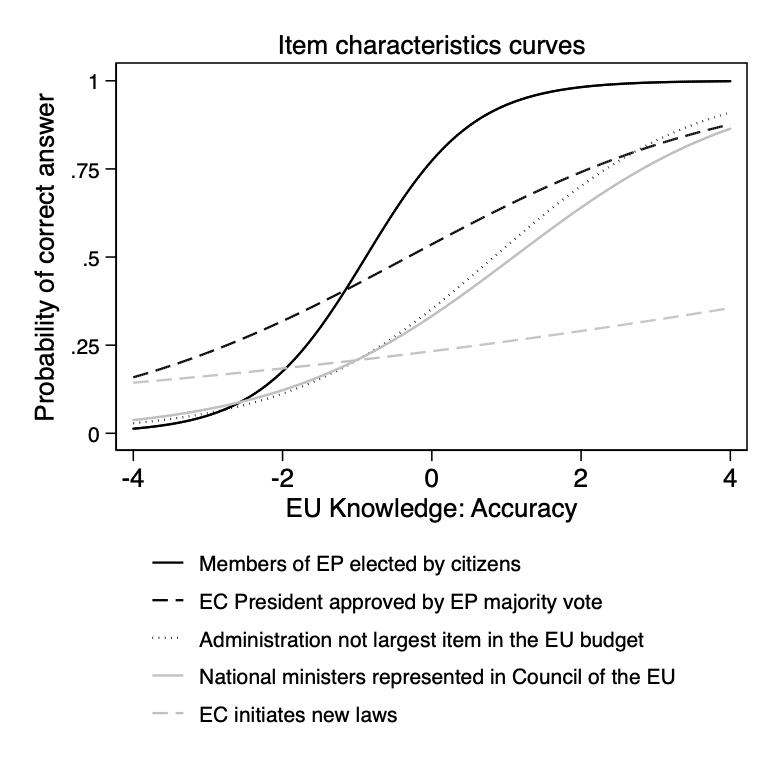 | 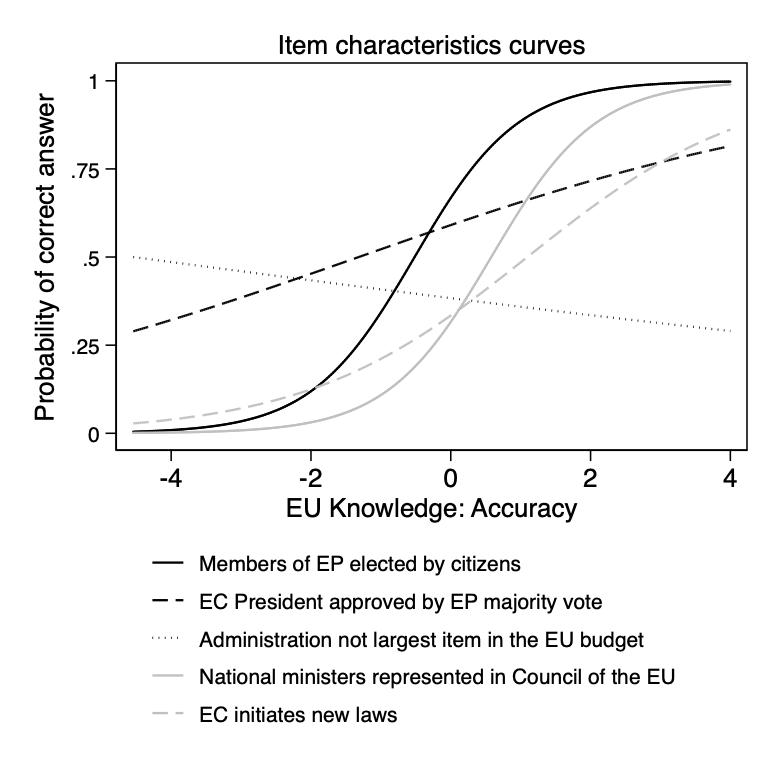 |

**Figure C3-2**: Correlation matrices - by country

| Germany | France | Spain | Hungary |
| --- | --- | --- | --- |
| 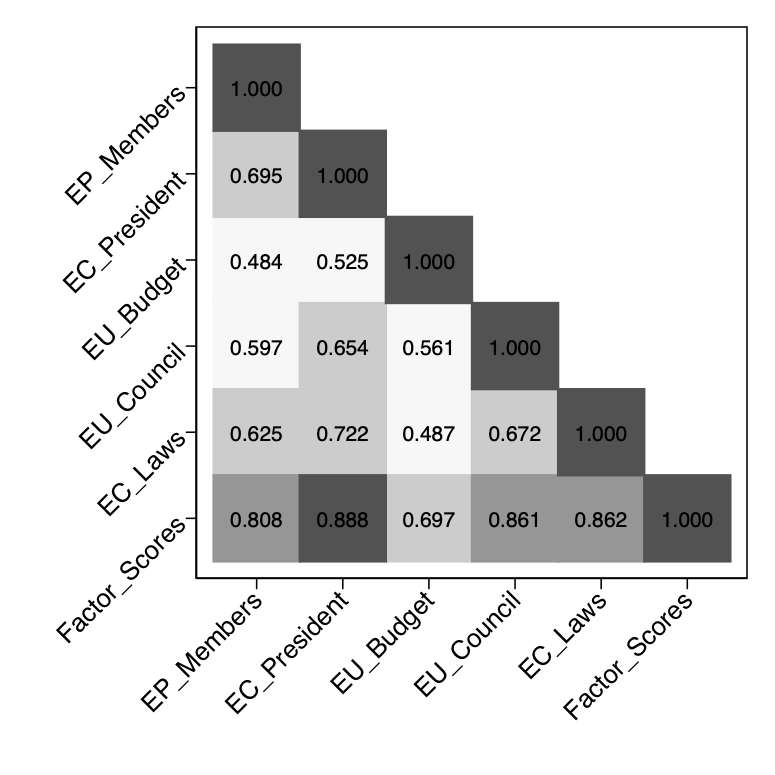 | 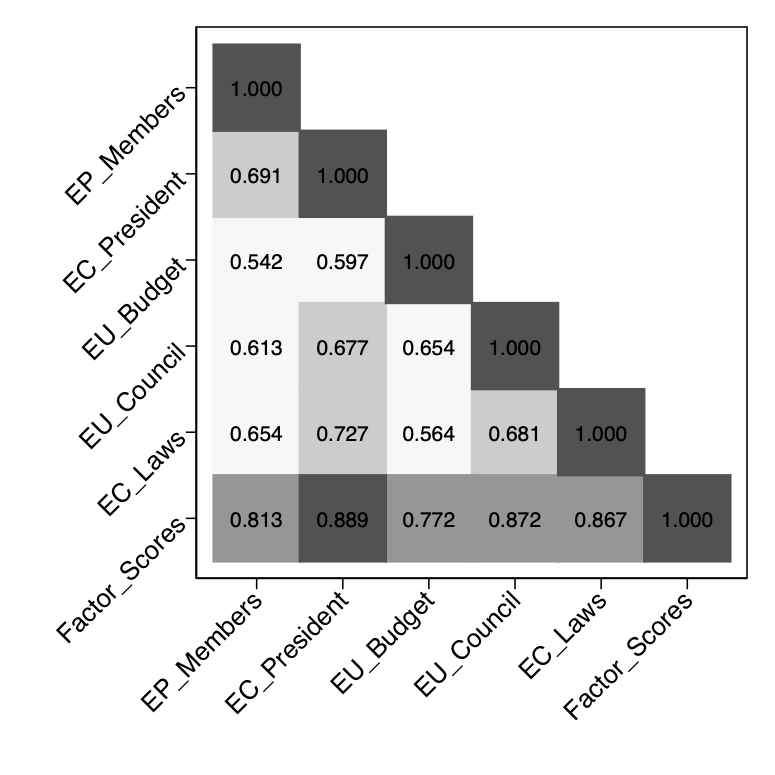 | 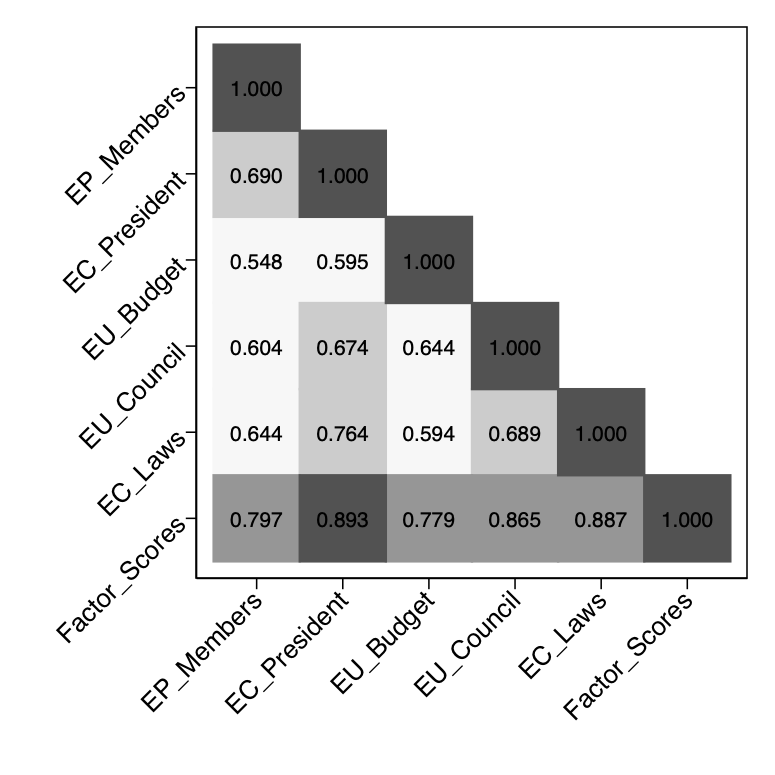 | 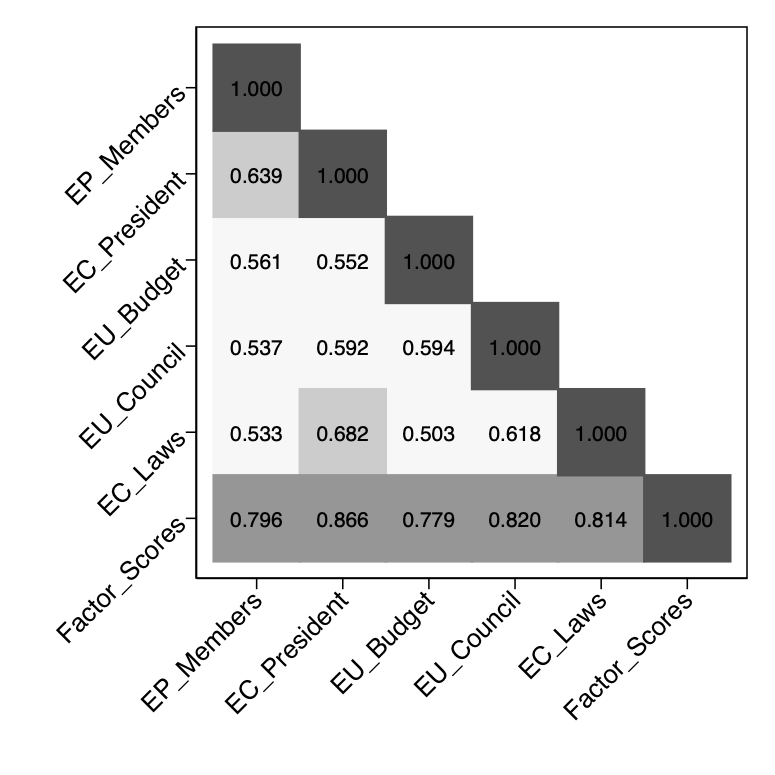 |
| Poland | Denmark | Italy | Austria |
| 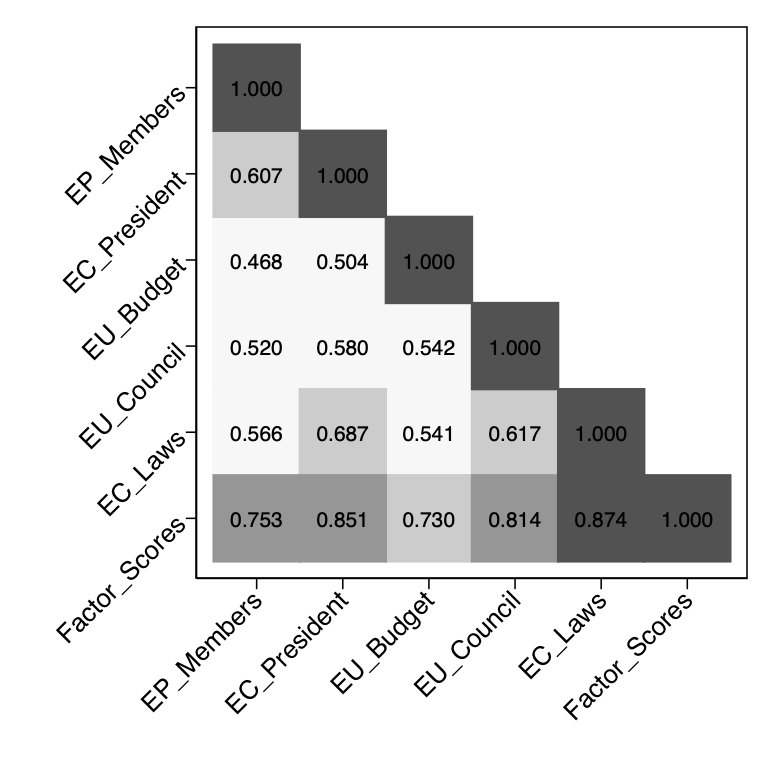 | 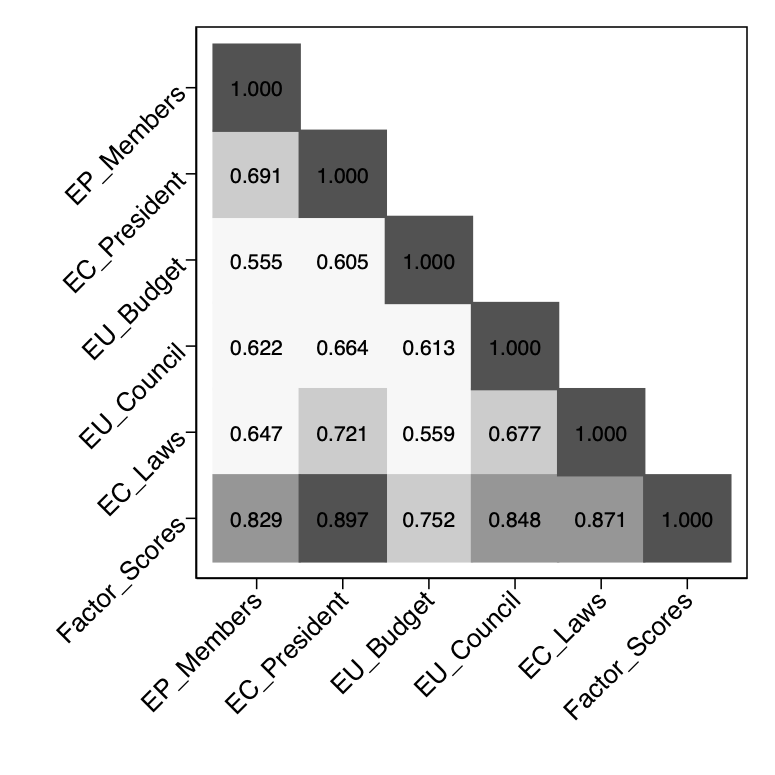 | 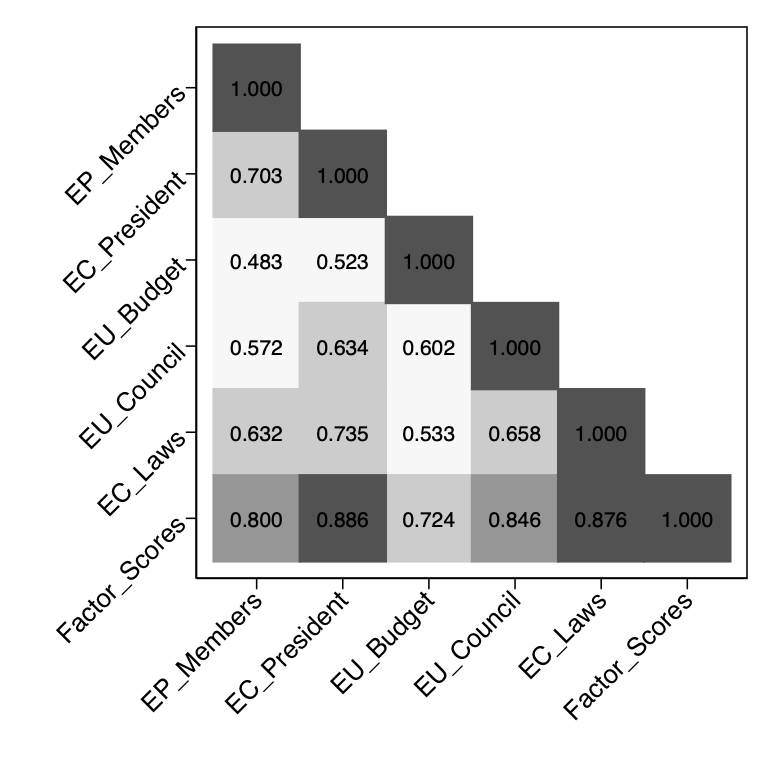 | 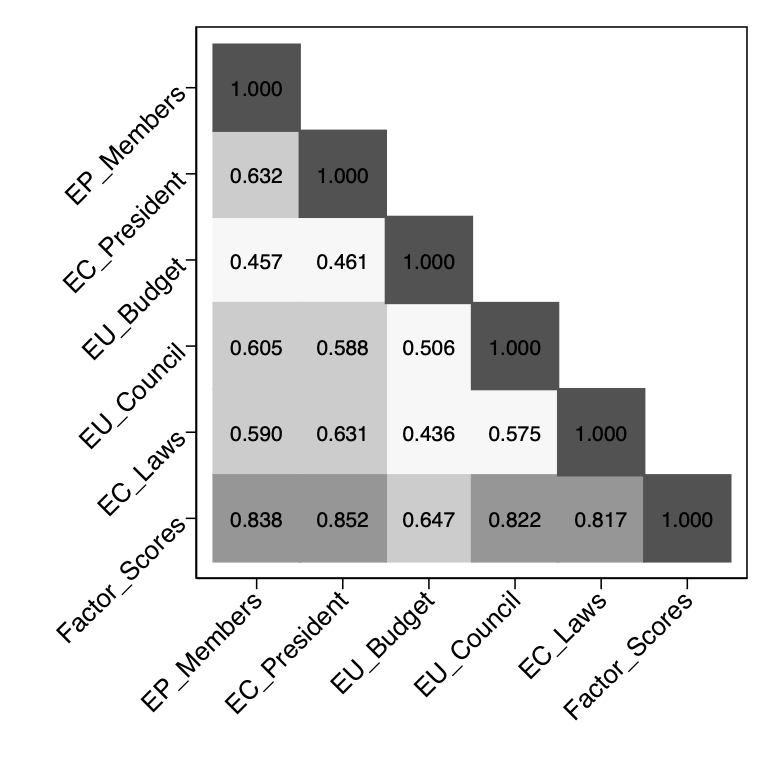 |

**Figure C3-3**: Heatmaps - by country

| Germany | France |
| --- | --- |
| 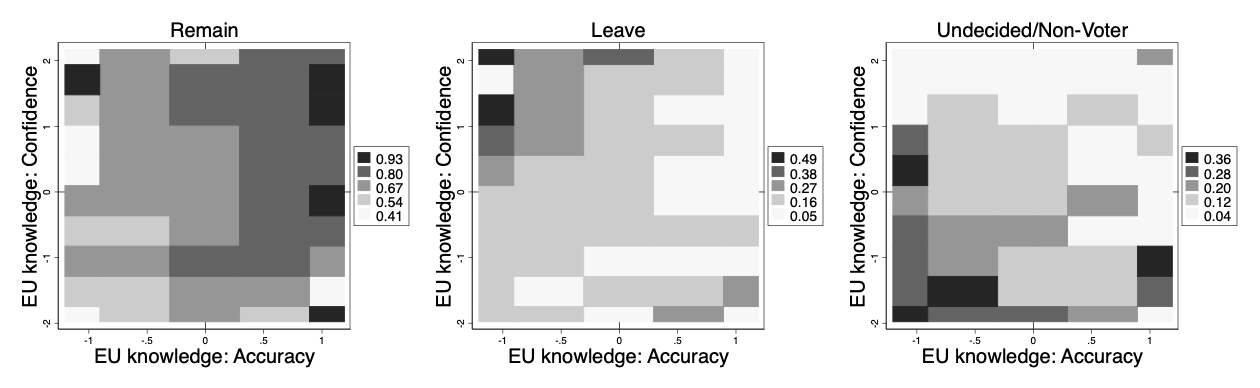 | 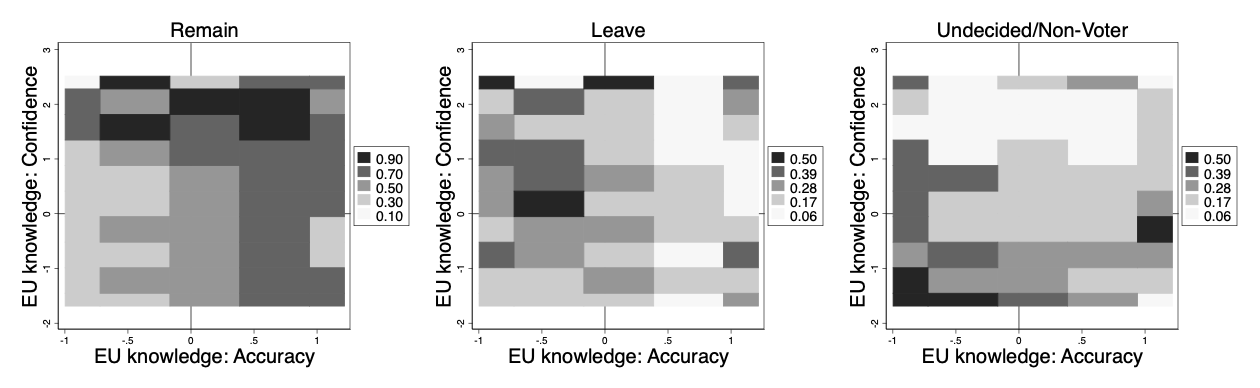 |
| Spain | Hungary |
| 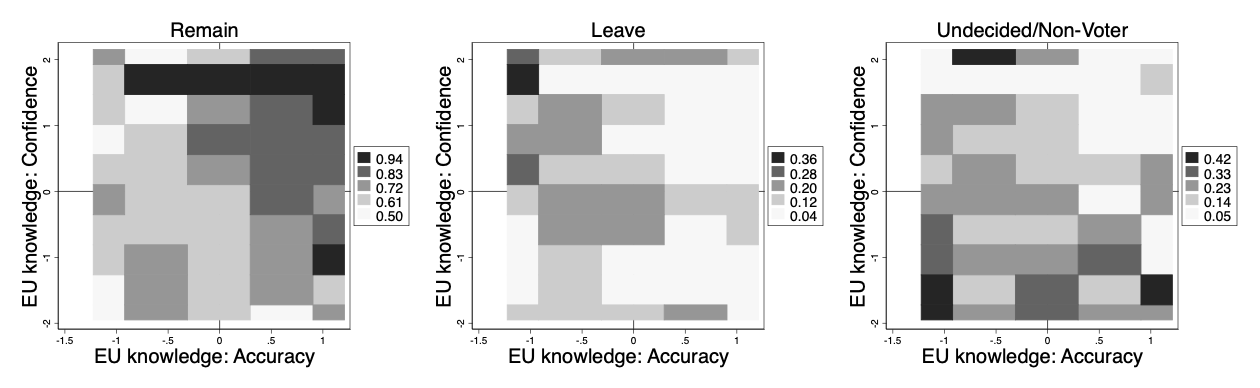 | 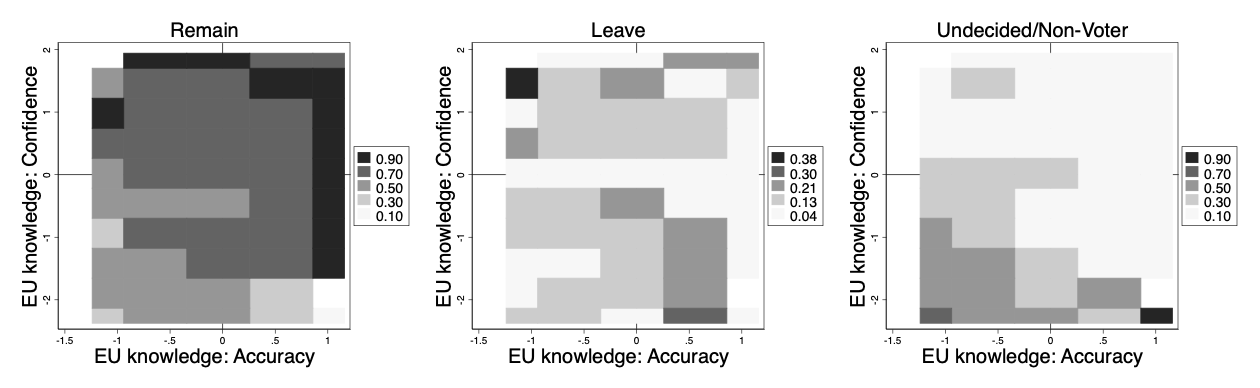 |
| Poland | Denmark |
| 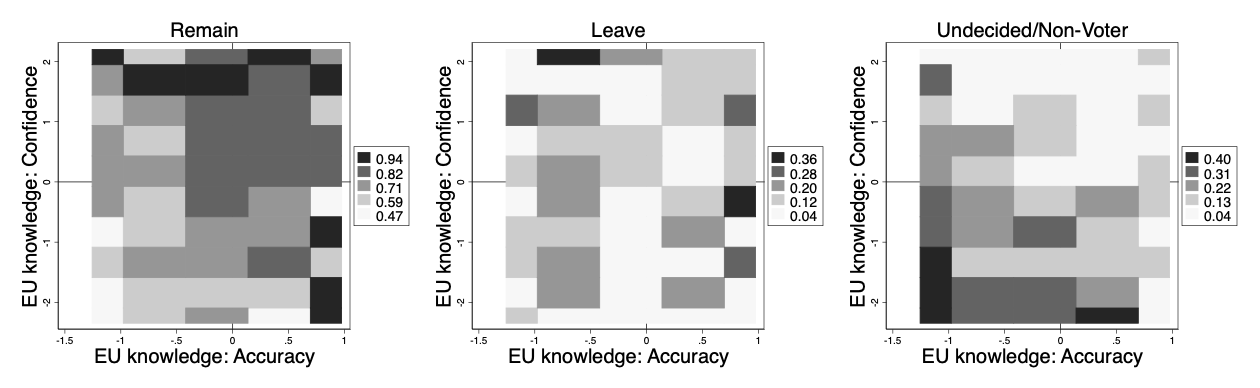 | 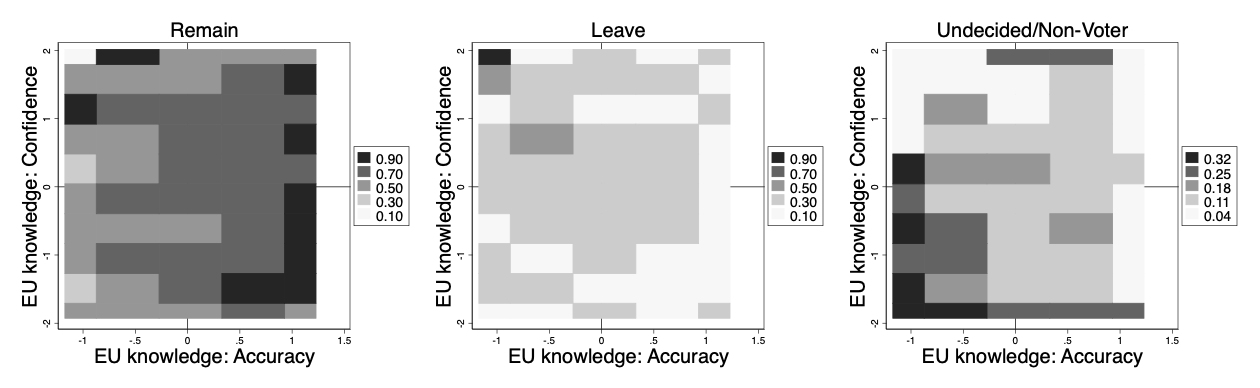 |
| Italy | Austria |
| 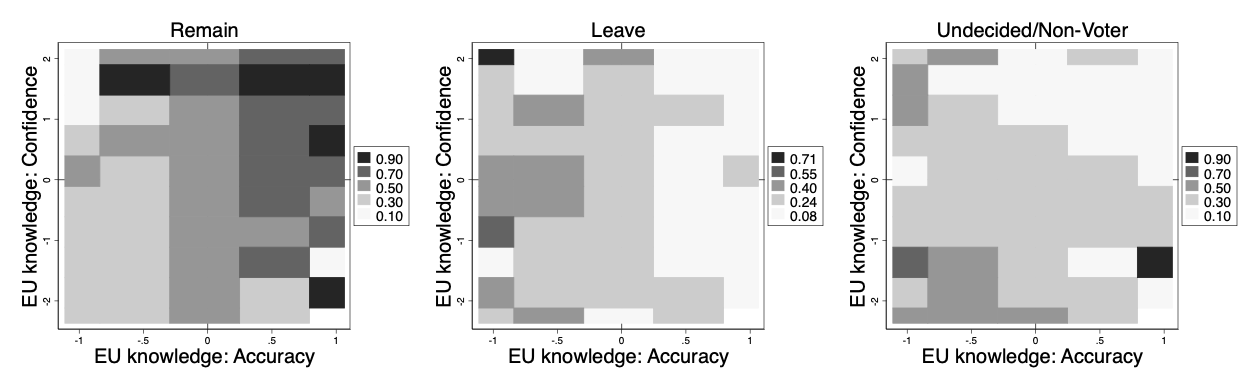 | 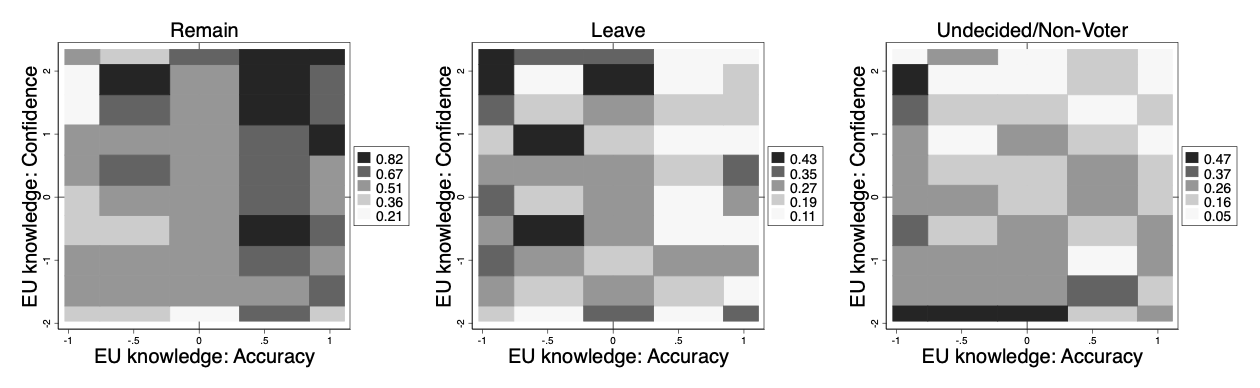 |

**Figure C3-4**: Average marginal effects of EU knowledge confidence - by country

| Germany | France |
| --- | --- |
| 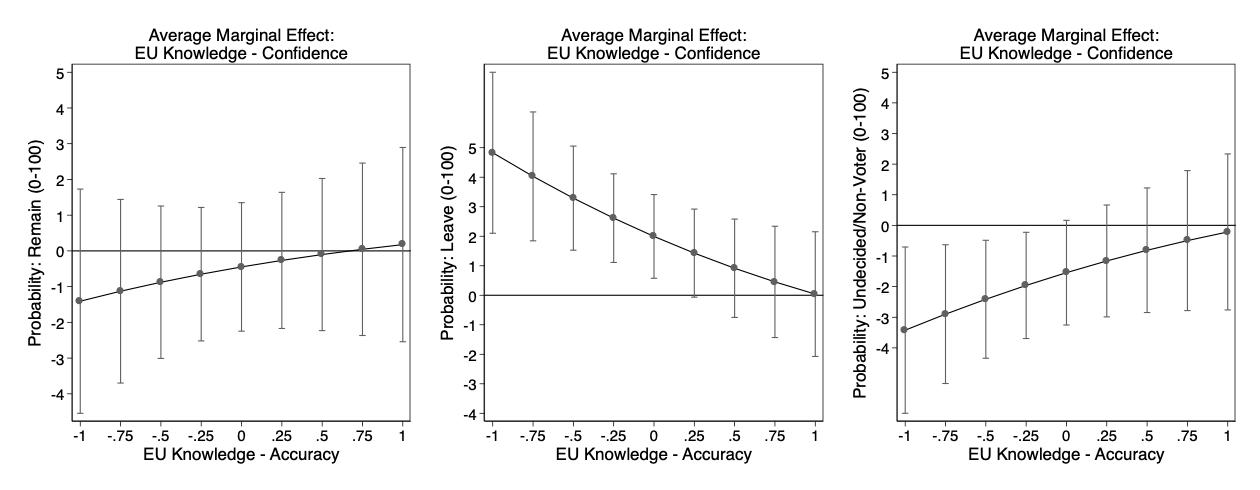 | 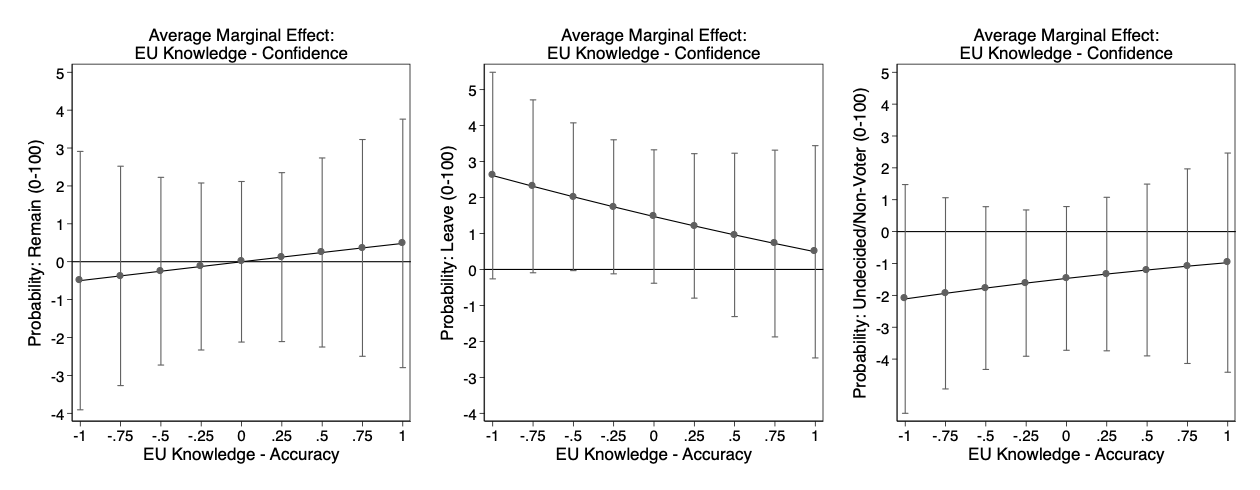 |
| Spain | Hungary |
| 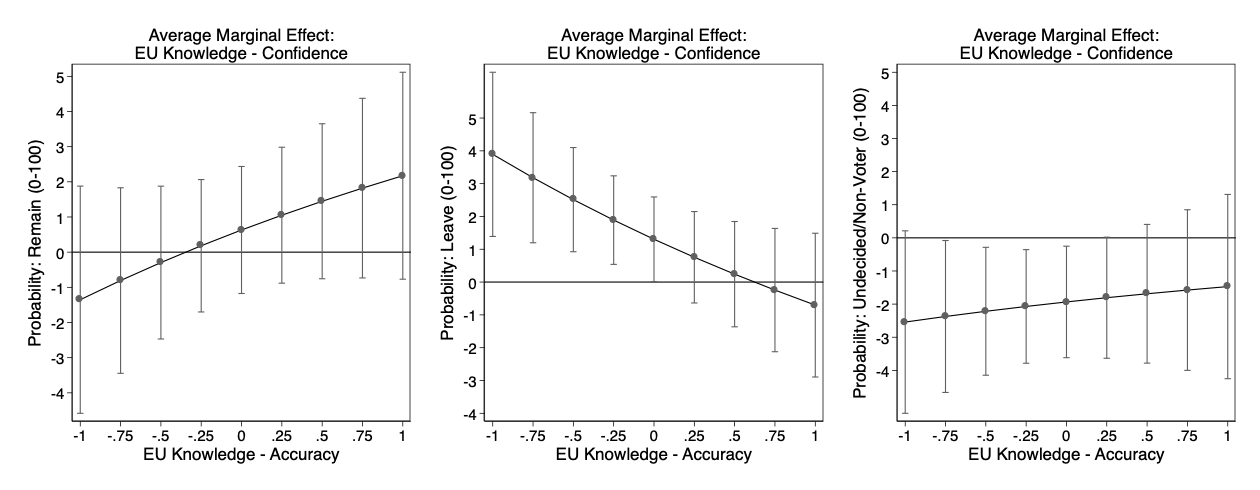 | 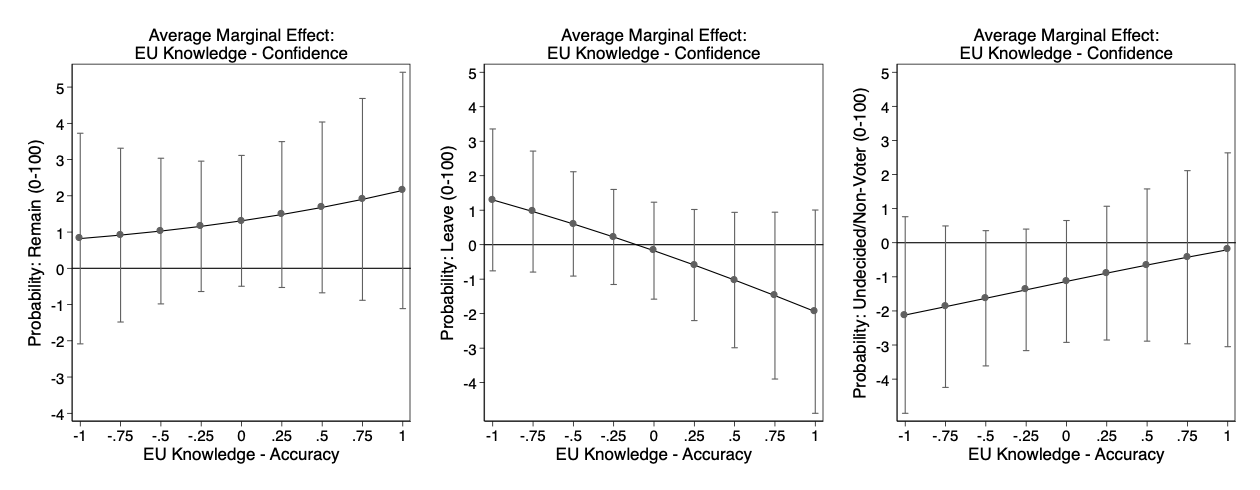 |
| Poland | Denmark |
| 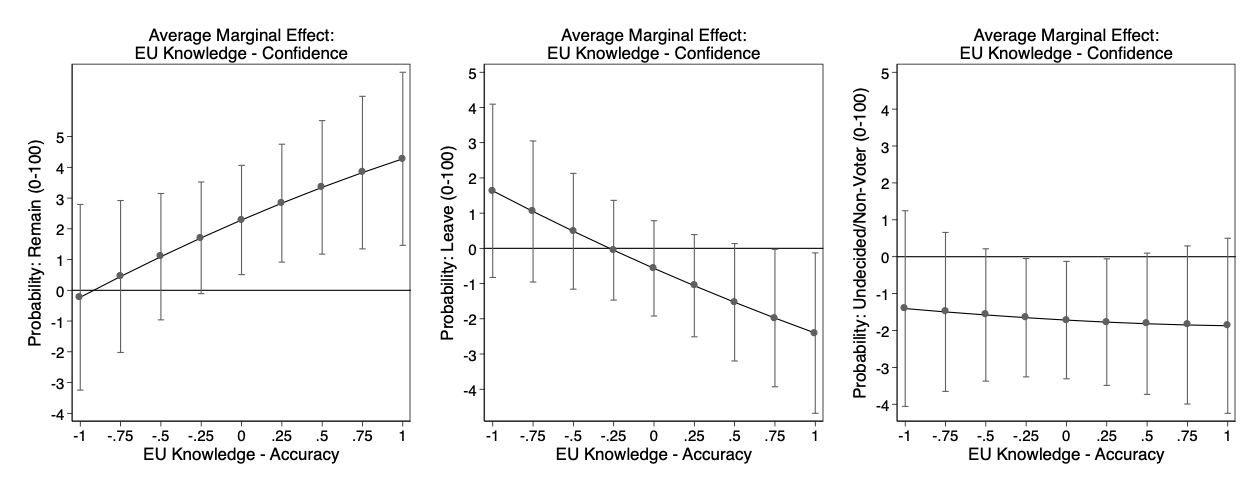 | 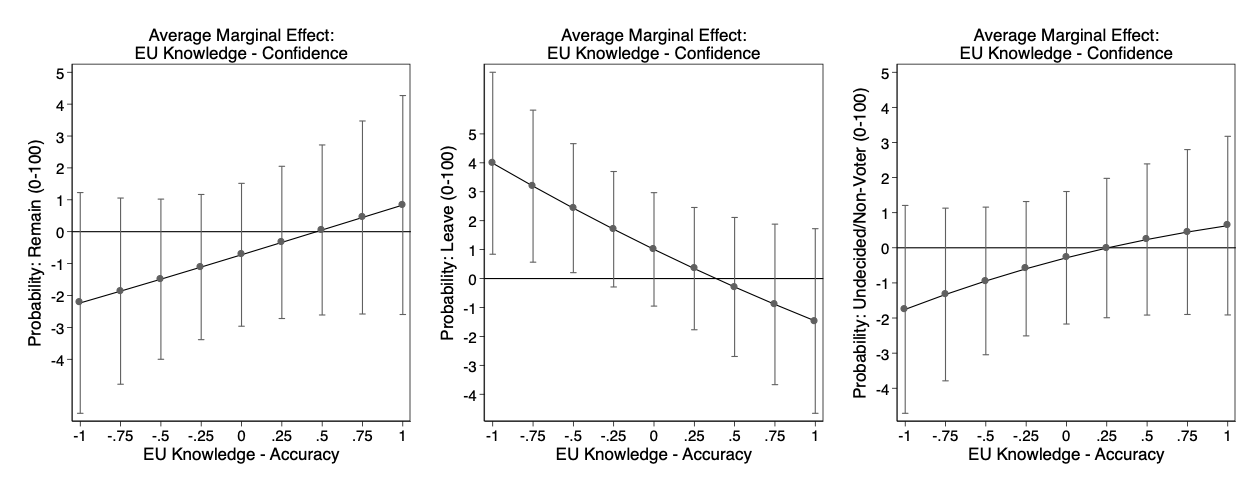 |
| Italy | Austria |
| 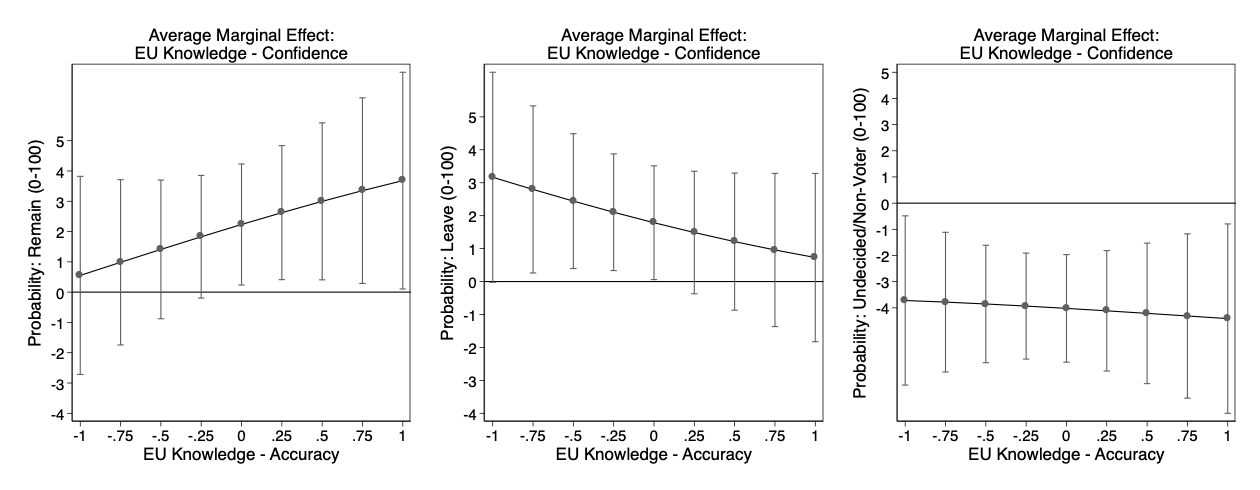 | 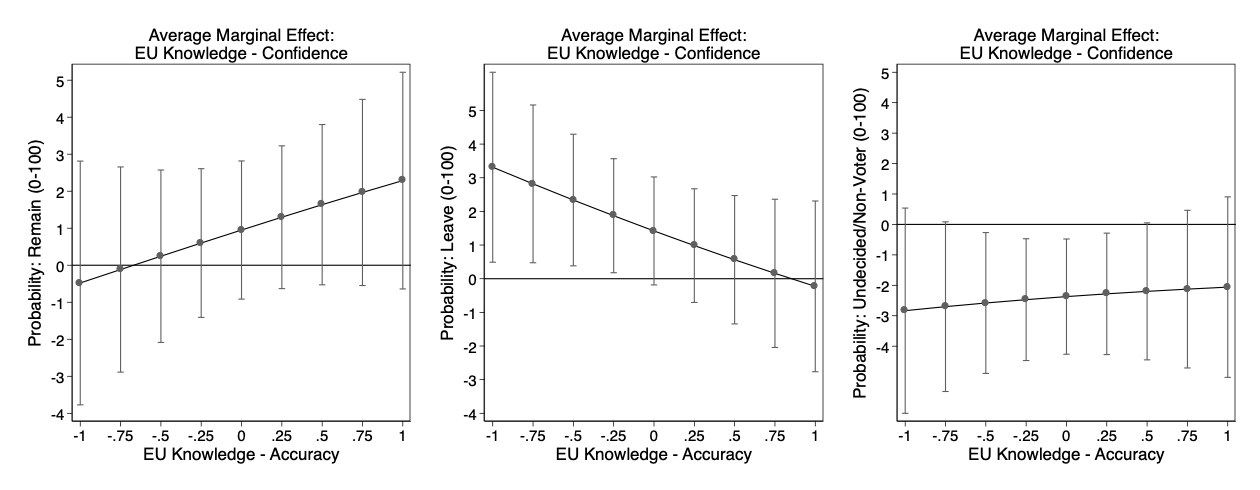 |

**Figure C3-5**: Average marginal effects of EU knowledge accuracy - by country

| Germany | France |
| --- | --- |
| 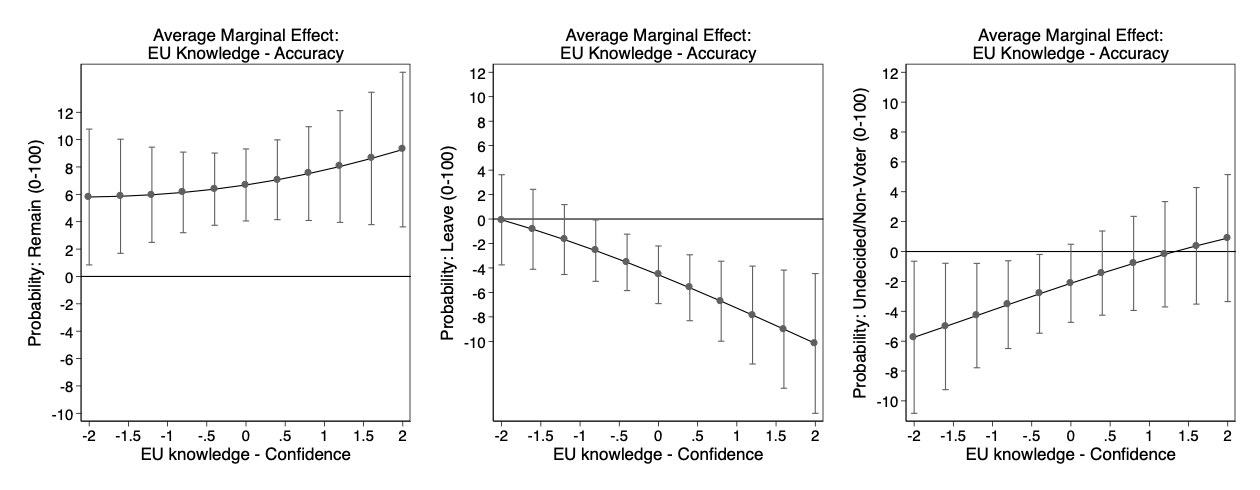 | 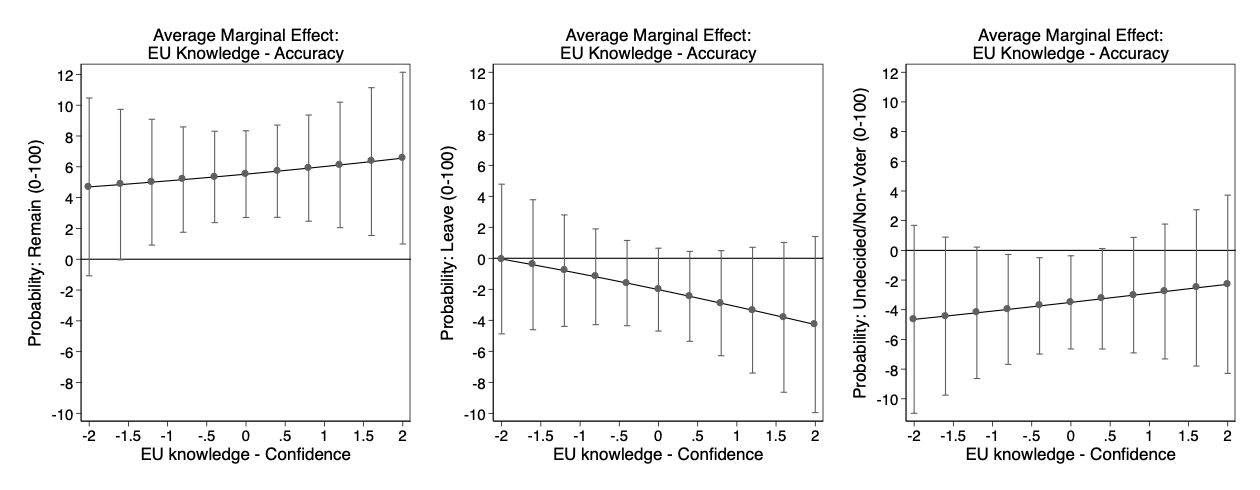 |
| Spain | Hungary |
| 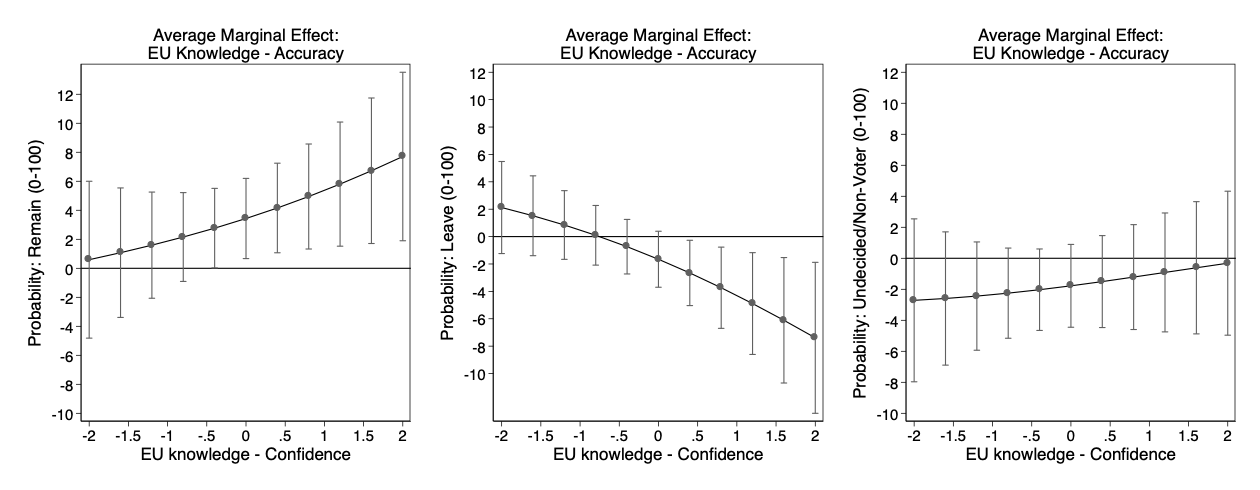 | 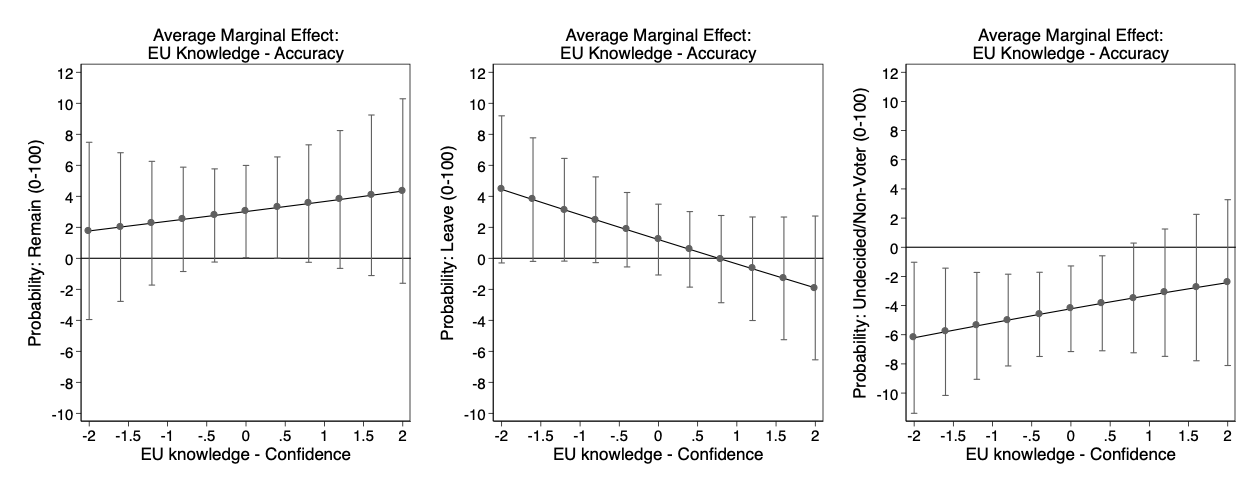 |
| Poland | Denmark |
| 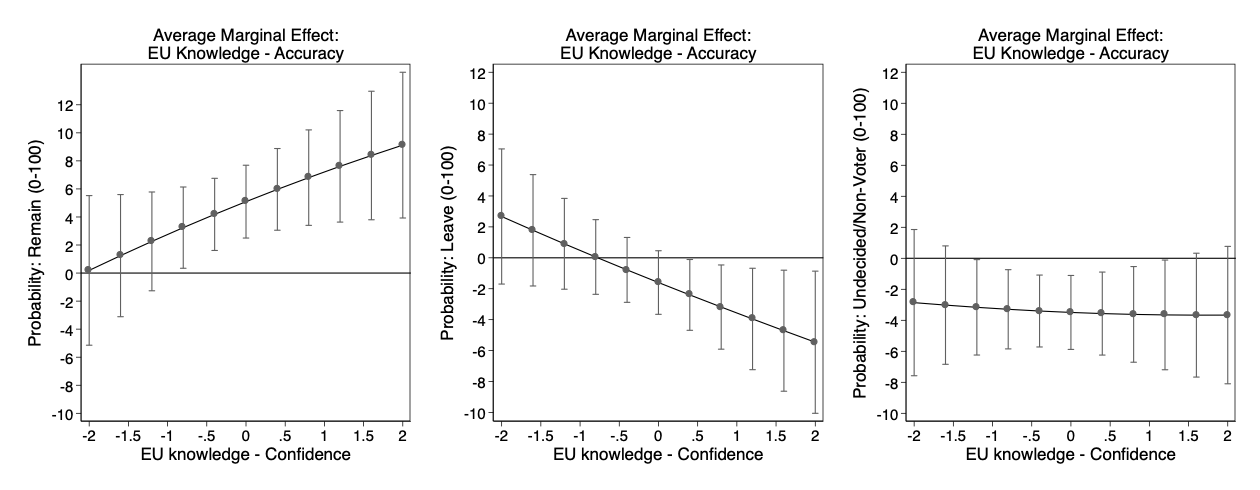 | 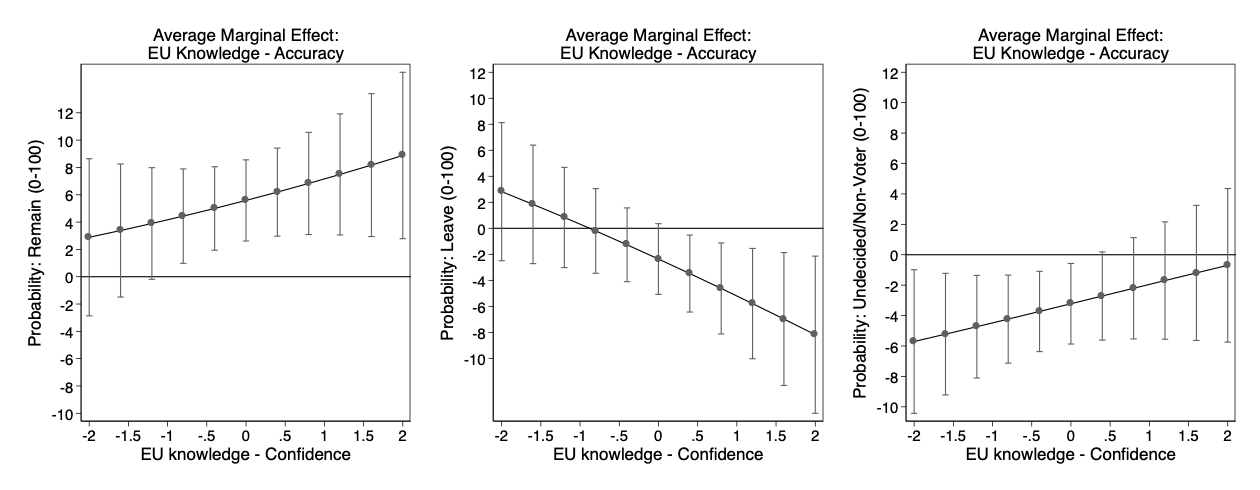 |
| Italy | Austria |
| 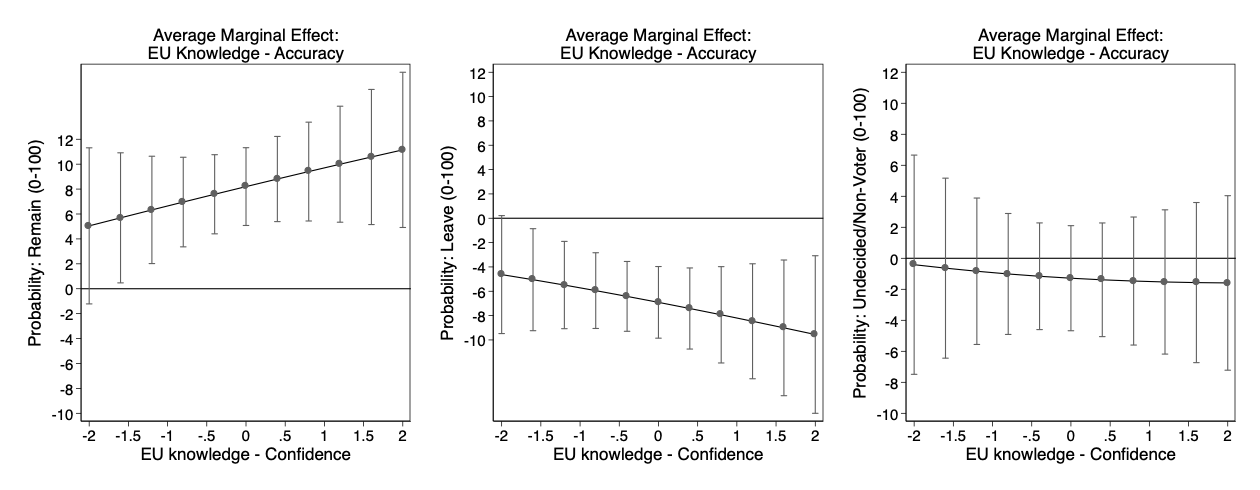 | 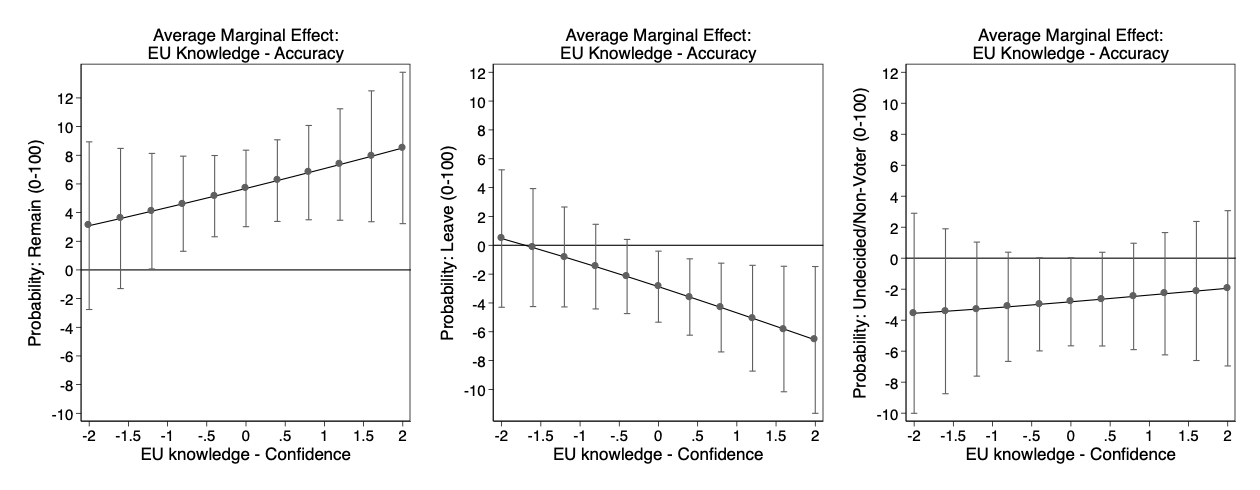 |

**Appendix D: Details on measurement scales**

Figure D1 shows the univariate distributions of the predicted scores for EU knowledge accuracy (left) and confidence (right). The values for accuracy are fairly normally distributed and range from slightly below to slightly above one, with an average of zero. As these values refer to the latent scale, they can only be interpreted as relative measures of the latent ability to provide a correct answer. As per the identifying assumption of the model, a unit shift on the latent scale is equal to a shift by one standard deviation of the latent dimension. Respondents at the low end are likely to answer all questions incorrectly, whereas respondents with high values are likely to answer all questions correctly. Please note, while the theoretical range of the latent variable as per model assumptions is naturally unbounded (ranging from minus to plus infinity), we can only observe the accuracy of knowledge over the range as captured by our items.

**Figure D1**: Univariate distribution of predicted scores for accuracy and confidence
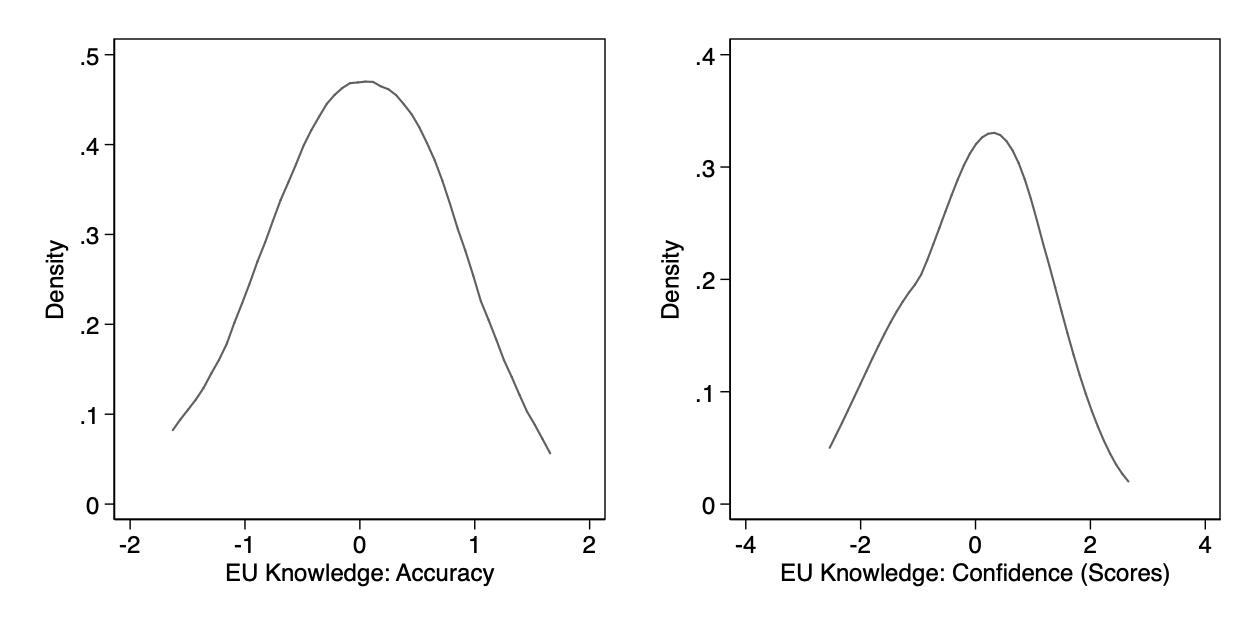


*Note*: Kernel density estimates (Epanechnikov kernel, bandwidth=0.5)

By construction of the measure, the predicted factor scores also have a mean of zero, reflecting average levels of confidence, and a standard deviation of 1. Again, the latent scale is essentially dimensionless and can therefore only be interpreted in relative (rather than absolute) terms. Moving by a unit of 1 implies moving by one standard deviation on the latent scale. Respondents at the low end of the scales show the lowest levels of confidence in their knowledge, whereas respondents at the high end are highly confident.

The advantage of using a factor analysis to create a scale of confidence is that it allows for evaluating the dimensionality of the single items. Based on the inspection of a scree plot, we find that the items load mainly on a single dimension (see Figure D2). This reflects the fact that the items correlate fairly strongly among each other in a manner explained by a single dimension of variance. Instead of using the predicted factor scores, another possibility to form a scale would have been to simply calculate the mean score across the items. The correlation between the factor scores and the mean score is 0.997^***^ (p<0.001). Therefore, it essentially makes no difference which approach is chosen to form the scale.

**Figure D2**: Scree plot and correlation matrix
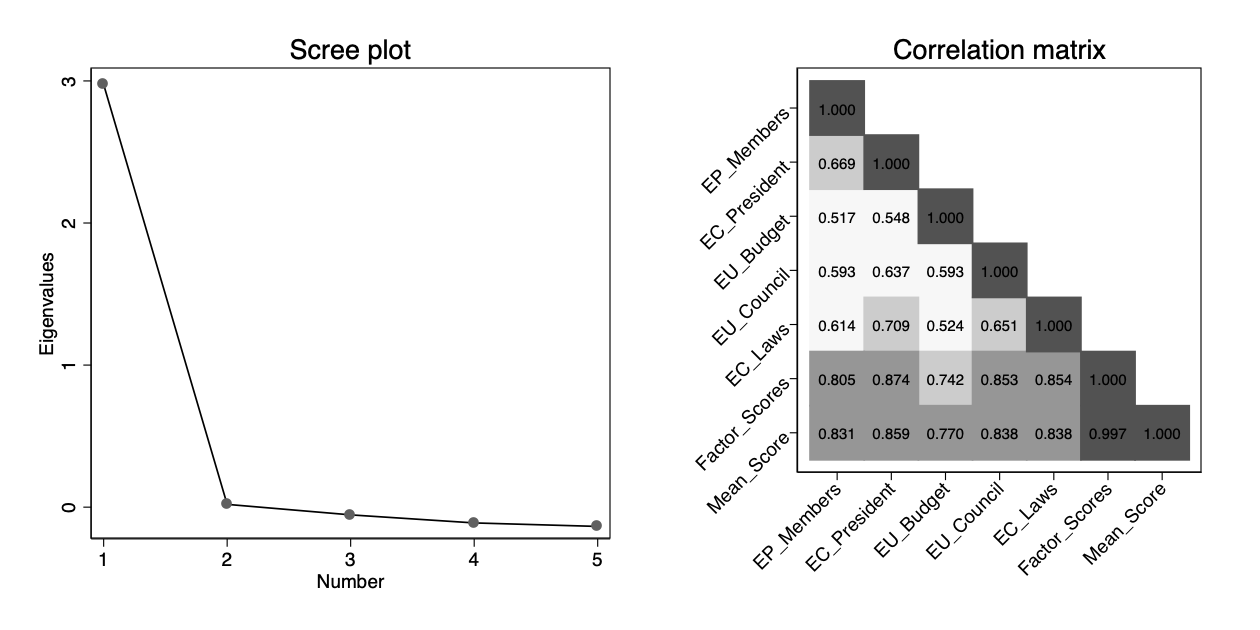


For completeness, we report additional details from the model output of the factor analysis in the tables below. As shown before in the scree plot, the eigenvalues drop down to near 0 after the first factor is retained (Table D1). If we nevertheless retain a second factor, we see that all items strongly load on the first factor and that the loadings are very low for the second factor (Table D2).

Table D1. Eigenvalues

| Factor | Eigenvalue | Difference | Proportion | Cumulative |
| --- | --- | --- | --- | --- |
| Factor1 | 2.973 | 2.954 | 1.104 | 1.104 |
| Factor2 | 0.020 | 0.074 | 0.007 | 1.112 |
| Factor3 | -0.054 | 0.057 | -0.020 | 1.092 |
| Factor4 | -0.111 | 0.025 | -0.041 | 1.050 |
| Factor5 | -0.135 | . | -0.050 | 1.000 |

*Note*: Method: principal factors. Retained factors: 2 (eigenvalues > 0).

Table D2. Factor loadings and unique variances

| Variable | Factor1 | Factor2 | Uniqueness |
| --- | --- | --- | --- |
| EP_Members | 0.758 | -0.035 | 0.425 |
| EC_President | 0.826 | -0.062 | 0.314 |
| EU_Budget | 0.677 | 0.091 | 0.534 |
| EU_Council | 0.784 | 0.066 | 0.381 |
| EC_Laws | 0.803 | -0.043 | 0.354 |

*Note*: Unrotated solution.

**Appendix E: Alternative representation of model predictions**

As continuous-by-continuous interactions in a multinomial logit model are difficult to interpret, we provide alternative representations of the Figure 6 and 7, relying on predicted probabilities instead of marginal effects. For this purpose, we first plot the predicted probabilities for accuracy at low (min.) and high (max.) levels of confidence in Figure E1, holding all other variables at observed (Hanmer and Kalkan 2013). We also plot the predicted probabilities for confidence at low (min.) and high (max.) levels of accuracy in Figure E2, again, holding all other variables at observed.

**Figure E1.** Predicted probabilities for accuracy at low and high levels of confidence


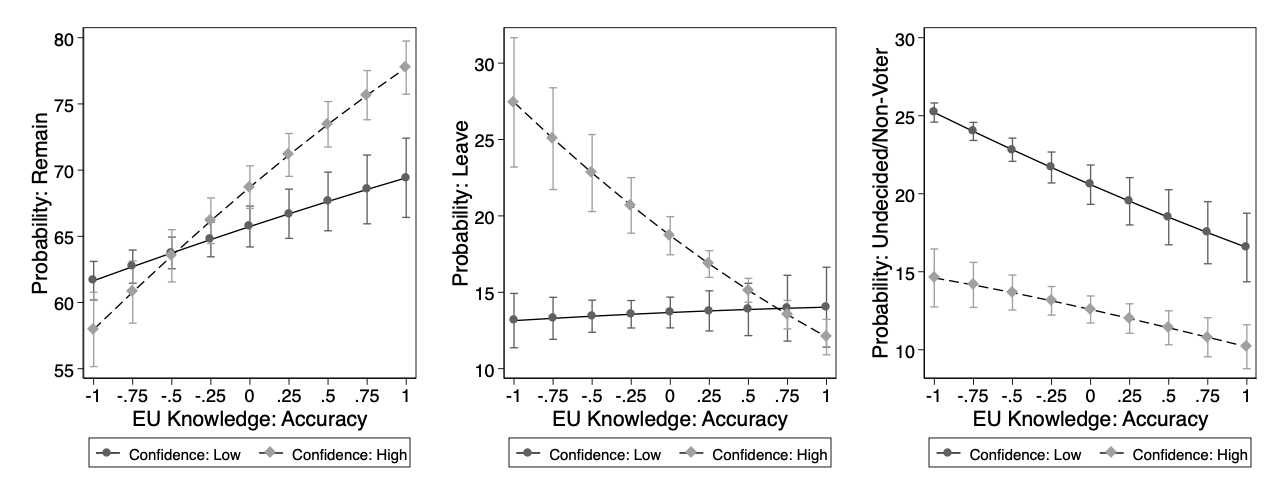


**Figure E2.** Predicted probabilities for confidence at low and high levels of accuracy


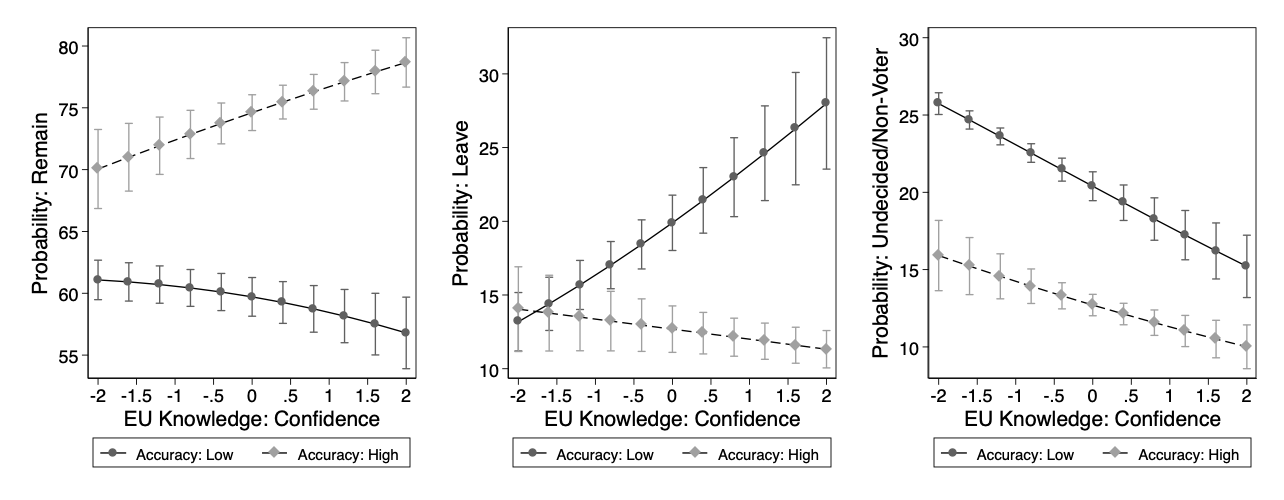


As can be seen from the left-hand panels in Figure E1 and E2, high accuracy of knowledge in conjunction with high levels of confidence in knowledge about the EU is positively related to voting to remain in a hypothetical referendum on EU membership (Hypothesis 1). Turning to mid panel, we see that low accuracy of knowledge in conjunction with high levels of confidence in knowledge about the EU is positively related to voting to leave in a hypothetical referendum on EU membership (Hypothesis 2). Lastly, looking at right-hand, we see that, regardless of the accuracy of knowledge, low confidence in knowledge about the EU is positively related to being undecided about remain or leave, or choosing to abstain in a hypothetical referendum on EU membership (Hypothesis 3). Hence, we arrive at the same conclusion that all three hypotheses are confirmed by the analysis. We hope that this additional visual representation of the model predictions helps to further clarify the implications of the results.

1. For classification, we rely here on the global mean of the pooled sample. [↑](#footnote-ref-1)
